# Adipocyte hypertrophy and lipid dynamics underlie mammary gland remodeling after lactation

**DOI:** 10.1038/s41467-018-05911-0

**Published:** 2018-09-04

**Authors:** Rachel K. Zwick, Michael C. Rudolph, Brett A. Shook, Brandon Holtrup, Eve Roth, Vivian Lei, Alexandra Van Keymeulen, Victoria Seewaldt, Stephanie Kwei, John Wysolmerski, Matthew S. Rodeheffer, Valerie Horsley

**Affiliations:** 1 Department of Molecular, Cellular and Developmental Biology, Yale University, 219 Prospect St., New Haven, CT 06520 USA; 2 Division of Endocrinology, Metabolism, and Diabetes, University of Colorado, Mail Stop F-8305; RC1 North, 12800 E. 19th Avenue P18-5107, Aurora, CO 80045 USA; 3 WELBIO, Interdisciplinary Research Institute (IRIBHM), Université Libre de Bruxelles (ULB), 808, route de Lennik, BatC, C6-130, 1070 Brussels, Belgium; 4 Department of Population Sciences and Bekman Institute, City of Hope, 1500 East Duarte Rd., Duarte, CA 91010 USA; 5 Section of Plastic and Reconstructive Surgery, Department of Surgery, Yale University, 333 Ceder St., New Haven, CT 06510 USA; 6 Section of Endocrinology and Metabolism, Department of Internal Medicine, Yale University, 333 Ceder St., New Haven, CT 06510 USA; 7 Department of Comparative Medicine, Yale University, 333 Ceder St., New Haven, CT 06510 USA; 8 Department of Dermatology, Yale University, 333 Ceder St., New Haven, CT 06510 USA

## Abstract

Adipocytes undergo pronounced changes in size and behavior to support diverse tissue functions, but the mechanisms that control these changes are not well understood. Mammary gland-associated white adipose tissue (mgWAT) regresses in support of milk fat production during lactation and expands during the subsequent involution of milk-producing epithelial cells, providing one of the most marked physiological examples of adipose growth. We examined cellular mechanisms and functional implications of adipocyte and lipid dynamics in the mouse mammary gland (MG). Using in vivo analysis of adipocyte precursors and genetic tracing of mature adipocytes, we find mature adipocyte hypertrophy to be a primary mechanism of mgWAT expansion during involution. Lipid tracking and lipidomics demonstrate that adipocytes fill with epithelial-derived milk lipid. Furthermore, ablation of mgWAT during involution reveals an essential role for adipocytes in milk trafficking from, and proper restructuring of, the mammary epithelium. This work advances our understanding of MG remodeling and tissue-specific roles for adipocytes.

## Abstract (web summary)

During mammary gland involution, the organ undergoes extensive remodeling. Here, the authors explore the role of mammary gland adipose tissue (mgWAT) in this process and demonstrate that adipocyte hypertrophy and lipid trafficking underlie mgWAT expansion and epithelial regression.

## Introduction

The mouse mammary gland (MG) epithelium develops and functions with an intimate connection to the surrounding adipose stroma^1–3^. Embryonic MG epithelial progenitor cells generate myoepithelial and luminal cells that form a branching ductal tree that expands to fill the stroma during puberty^4^. The mammary stroma is composed primarily of mature adipocytes, which we identify here as the MG white adipose tissue (mgWAT) depot. During pregnancy, luminal epithelial cells undergo terminal differentiation to form alveolar epithelial cells (AECs) that produce and secrete milk proteins, carbohydrates,  and lipids during lactation. Lactation stimulates stromal adipocytes to delipidate almost entirely, initially providing secretory AECs a local source of lipid for milk fat production^1,5–8^. Weaning triggers involution, during which the epithelium regresses and mgWAT expands rapidly to repopulate the stroma, becoming as prominent in size and adipocyte-specific gene expression as in the MG during pregnancy^9,10^.

The cellular events of involution occur in two phases: an initial, reversible phase occurring in the first three days, during which programmed cell death (PCD) is initiated in epithelial cells; and a second, irreversible phase occurring predominantly between days 4 and 7 of involution, during which AECs undergo a second wave of PCD, the alveoli collapse, and remodeling occurs^9,11^. The majority of tissue re-organization is complete in the first week of involution and is essential for proper remodeling of the mammary epithelium in preparation for possible subsequent rounds of lactation^9^. While the mgWAT depot is required for normal development of the mammary epithelium^1–3,5,12,13^, how adipocytes re-emerge and support epithelial remodeling after lactation is not fully understood.

WAT can regress and expand dynamically in response to various stimuli and physiological scenarios, including starvation, obesogenic diet, cold stress, dermal infection and wound healing, irradiation and chemotherapy, and hair cycling^14–25^. However, the in vivo cellular and molecular mechanisms that control WAT growth and regression are not well understood. We and others have recently identified cells and molecules that control depot-specific adipose tissue growth^14–24^. Since mature adipocytes are post-mitotic^26^, expansion of most WAT depots occurs through two mechanisms^27,28^: (1) Adipogenesis, or the generation of new mature adipocytes: a multi-step process that involves the proliferation of adipocyte precursors (APs), cell cycle exit, cellular differentiation, and hypertrophy of newly formed mature adipocytes as they fill their lipid droplet with triglycerides; or (2) Hypertrophy of existing adipocytes through lipid production and/or uptake. Whether one or both of these processes contribute to mgWAT expansion during involution is unknown.

To identify the cellular mechanisms underlying mgWAT expansion during involution, we employed several techniques to examine the distinct fates of epithelial and adipocyte lineages in the MG during involution. We define resident APs in the MG stroma in mice and humans, and characterize a population of small mature adipocytes retained in the gland throughout lactation. Data from in vivo proliferation assays, pharmacological inhibition of adipogenesis, long-term genetic lineage tracing, and teat sealing experiments reveal that locally controlled hypertrophy of existing adipocytes is a major mechanism of adipocyte repopulation during MG involution. We develop an in vivo lipid tracking assay used in combination with lipidomic analysis of MG adipocyte fatty acids (FAs) to show that adipocytes fill with epithelial-derived milk lipid as they undergo hypertrophy. Finally, we establish a method to specifically ablate adipocytes in mgWAT immediately prior to MG involution to identify that adipocytes are necessary for proper epithelial remodeling. Our study identifies key roles for adipocytes during involution in regenerating the mammary stroma via hypertrophy, facilitating the transfer of remaining milk lipid into the stroma at the conclusion of lactation, and supporting epithelial regression.

## Results

### Mature adipocytes increase in size throughout involution

**Fig. 1:**
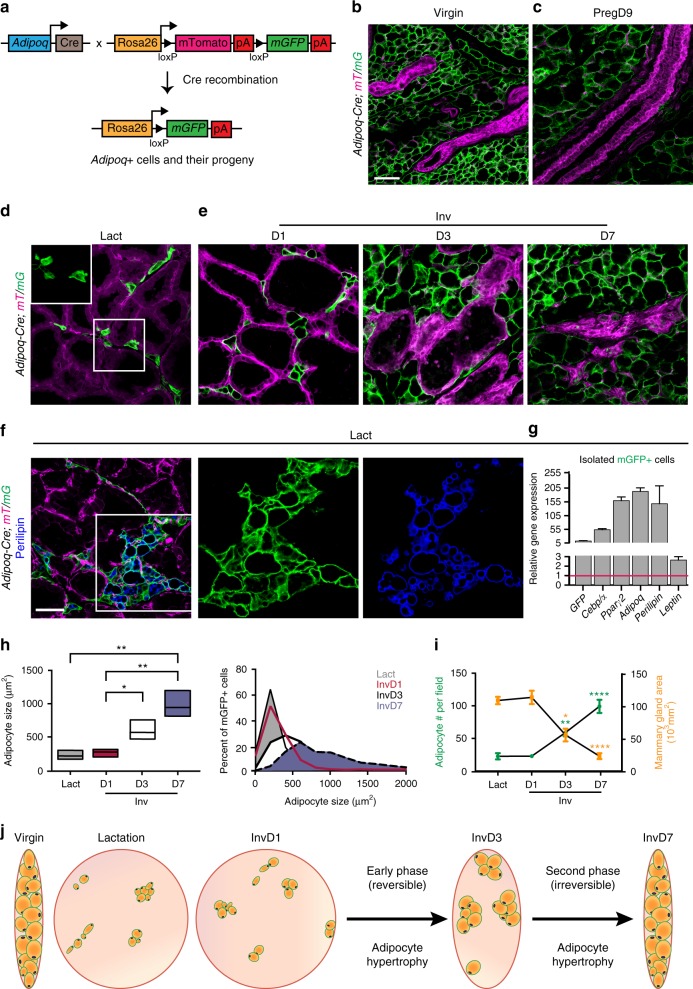
Adipocyte and MG dynamics throughout the lactation cycle. **a** Schematic summarizing genetic strategy to express mGFP in mature adipocytes. **b**–**f** Representative images of adipocytes (green) and all other cell types (magenta) in MGs from *Adipoq-Cre; mT/mG* mice at a mature, virgin stage (**b**), pregnancy day 9 (**c**), lactation day 10 (**d**, **f**), and involution days 1, 3, and 7 (**e**). Scale is equivalent in **b**–**e**. Tissue in **f** is also stained with anti-perilipin antibodies. **g** Representative qPCR results showing expression of mature adipocyte-associated genes in small mGFP+ cells isolated from lactating MGs normalized to total stromal vascular fraction. **h** Average (left panel) and distribution (right panel) of the size (cross-sectional area) of mGFP+ cells at the indicated time points. *n* ≈ 900 adipocytes in three mice for each time point. **i** Average number of adipocytes per field (left axis) and area of a 2D cross section of the MG (right axis) at the indicated time points. *n* ≈ 15–315 fields or one 2D cross section in each of 3–5 mice. **j** Schematic summarizing changes during the lactation cycle to the size of the MG, and size and distribution of adipocytes. Error bars represent mean ± SEM. Significance was calculated using a two-tailed unpaired *t*-test to compare individual time points (**h**) or one-way ANOVA with Tukey’s multiple comparison test (**i**). *P < 0.05, **P < 0.01, ****P < 0.0001. Bounds of box plot (**h**) reflect data min and max with centre line at the mean. White boxes indicate insets and scale bars are 50 μm. Preg pregnancy, Inv involution, Lact lactation, D day, *mT/mG* membrane tomato/membrane GFP, MG mammary gland, n.s. not significant

To characterize mature adipocytes in the MG stroma, we used a mouse strain with a dual fluorescent membrane-localized tdTomato/eGFP (*mT/mG*) reporter, which marks Cre-mediated excision by a heritable switch from mTomato expression to mGFP expression^29^. We crossed these mice to a line expressing Cre recombinase driven by the *Adipoq* promoter to visualize mature adipocytes^30^ (Fig. 1a). MGs of the resulting *Adipoq-Cre; mT/mG* virgin mice displayed bright mGFP expression in adipocytes surrounding the mTomato+, GFP− epithelial ducts (Fig. 1b).

To define the dynamics of mature adipocytes during reproduction, we analyzed sections of MGs from *Adipoq*-Cre; *mT/mG* mice during pregnancy, lactation, and involution. As in virgin MGs, large mGFP+ cells occupied the majority of pregnant mammary stroma (Fig. 1c). While few adipocytes were unambiguously recognizable in the lactation-stage MG stroma in hematoxylin and eosin (H&E)-stained sections (Supplementary Fig. 1a), small mGFP+ cells were clearly visible in the stroma of lactating MGs of *Adipoq*-Cre; *mT/mG* mice (Fig. 1d). During involution, as mTomato+ alveolar ducts regressed, mgWAT expanded, as marked by an increased area of the MG occupied by mGFP+ cells (Fig. 1e). Consistent with the expression of *Adiponectin*, 99.7% of mGFP+ cells expressed the mature adipocyte-associated lipid protein perilipin^16,30,31^ at involution day 7 (Supplementary Fig. 1b). Small mGFP+ cells in the MG during lactation also stained positively for perilipin (Fig. 1f), and when purified from the MG at this stage, expressed mRNA for markers of mature adipocytes including *Cebp/α*, *Pparγ2*, *Adipoq*, *Perilipin*, and *Leptin*^30^ (Fig. 1g). Thus, small mature adipocytes exist in the MG during lactation.

We next focused on involution to examine hypertrophy of existing adipocytes. Morphometric analysis of adipocyte size in sections of lactation- and involution-stage MGs (Fig. 1d, e) enabled measurement of cells as small as 40 µm^2^, and revealed that individual mature adipocytes increase 4.1-fold in cross-sectional area (CSA) throughout involution (Fig. 1h), with significant CSA increases observed at involution days 3 and 7 compared to day 1. This observation is consistent with the increasing size of perilipin+ lipid droplets in MG adipocytes throughout involution (Supplementary Fig. 1c, d) and point to robust adipocyte hypertrophy during both the initial (reversible) and second (irreversible)^11^ phases of involution.

To begin to examine adipocyte cell numbers during involution, we quantified the number of mature adipocytes in sections of MGs between lactation and involution. While the average number of adipocytes per field in MG sections increased significantly from lactation to involution day 7 (Fig. 1i), the overall size of isolated MGs decreases markedly during this same time frame (Supplementary Fig. 1e), as shown by the 4.7-fold decrease in the CSA of MG sections between lactation and involution day 7 (Fig. 1i and Supplementary Fig. 1f). These changes are summarized in Fig. 1j: while an increasing number of adipocytes is observed in MG sections during involution (Fig. 1e, i), there is also an increased density of adipocytes as the overall size of the MG shrinks (Fig. 1i and Supplementary Fig. 1e, f).

### Adipocyte–epithelial transdifferentiation was not detected

**Fig. 2:**
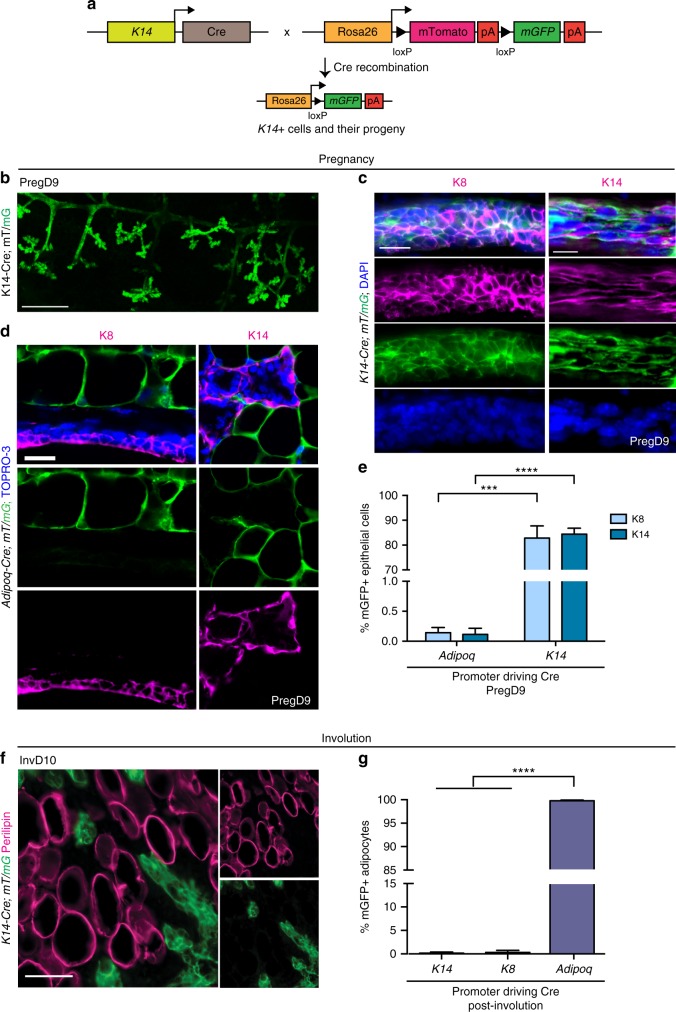
Reversible transdifferentiation is not the major mechanism of mgWAT expansion. **a** Schematic summarizing genetic strategy to drive mGFP expression in K14-expressing cells and their progeny, and mTomato expression in all other cells. **b** Whole-mount MG tissue imaged at pregnancy day 9 in *K14-Cre; mT/mG* mice. Three-dimensional projection of tile scan image representing 30–40 *z*-stacks. Scale bar is 500 μm. **c**, **d** MGs from *K14-Cre; mT/mG* (**c**) or *Adipoq-Cre; mT/mG* (**d**) mice were stained with antibodies against K8 and K14 to mark luminal epithelial and myoepithelial cell lineages, respectively, and indicated nuclear dyes. **e** Quantification of the percentage of K8+ or K14+ epithelial cells that are mGFP+ in *Adipoq-Cre; mT/mG* or *K14-Cre; mT/mG* mice at Pregnancy day 9. *n* = 3 (K8) or 4 (K14) mice for both genotypes. 575-3136 cells were analyzed. **f** Representative image of MG from *K14-Cre; mT/mG* mouse at involution day 10 immunostained for perilipin. **g** Quantification of the percentage of mGFP+, Perilipin+ adipocytes in *K14-Cre; mT/mG; K8-CreER; Rosa-YFP*, or *Adipoq-Cre; mT/mG* mice after involution. *n* = 3 mice, for which 1039–3359 adipocytes were analyzed for each genotype. Data are mean ± SD. Scale bars represent 50 μm in **c** and **f**, and 20 μm in **d**. Significance was calculated using a two-tailed unpaired *t*-test (**e**) and one-way ANOVA with Tukey’s multiple comparison test (**g**). ***P < 0.001, ****P < 0.0001. Preg pregnancy, Inv involution, Lact lactation, D day, *mT/mG* membrane tomato/membrane GFP, DAPI 4′,6-diamidino-2-phenylindole, K keratin

We next examined whether new adipocytes may be generated during involution. Adipocytes in the MG have been reported to generate luminal epithelial cells during pregnancy and to derive from luminal epithelial cells after involution^32,33^. Although prior studies used cytoplasmic β-galactosidase genetic models to trace the lineage of adipocytes in the MG^32,33^, the mT/mG reporter is a more precise method to label adipocytes which lack a large cytoplasm^30^. Therefore, we generated *Keratin 14* (*K14)-Cre; mT/mG* mice, which during development label progenitors of all MG epithelial cell types—both myoepithelial and luminal epithelial cells^34,35^—with mGFP (Fig. 2a). Whole-mount images of large regions of the MG from *K14-Cre; mT/mG* mice during pregnancy revealed that the majority of epithelial ducts express mGFP (Fig. 2b). During pregnancy, 85.9% of K8+ luminal and 87.9% of K14+ myoepithelial lineages expressed mGFP in these mice (Fig. 2c, e), which is consistent with previous studies demonstrating that both epithelial lineages are traced from K14-expressing embryonic precursors^34^.

We then immunostained MG sections with antibodies against K8 and K14 to analyze epithelial cells in pregnant *Adipoq-Cre; mT/mG* mice. Unlike the adipocyte-binding protein 2 (aP2)-Cre mouse line previously used to trace adipocytes in the MG^33^, but which also traces several other cell types^36,37^, mature adipocytes in *Adipoq-Cre; mT/mG* mice are specifically, stably, and efficiently labelled in mature, non-pregnant mice^30,37^ (Fig. 1b). During pregnancy, mGFP+ luminal (K8+) or myoepithelial (K14+) cells were not detected in MGs from *Adipoq*-*Cre; mT/mG* mice (<0.5% in each lineage) (Fig. 2d, e). Thus, in contrast to previous reports^32,33^, mature adipocytes are not a major source of precursors for MG epithelial cells during pregnancy.

Next, we examined whether either epithelial cell lineage, traced by mGFP in *K14-Cre; mT/mG* mice in pregnancy (Fig. 2b, c, e), contributes to the expansion of adipose tissue during involution. In contrast to the ~100% labeling of adipocytes in *Adipoq-Cre; mT/mG* mice at involution day 7 (Fig. 1e, Supplementary Fig. 1b), only 0.25% of adipocytes were labeled with mGFP in *K14-Cre; mT/mG* mice as visible in MG whole mounts which lack mGFP+ cells that morphologically resemble adipocytes (Supplementary Fig. 2a), and quantified in perilipin antibody-immunostained MG sections (Fig. 2f, g).

To further examine whether luminal epithelial cells form adipocytes during involution, we utilized *Keratin 8 (K8)-CreER; Rosa-YFP* mice, in which a single low dose of tamoxifen administered to 1-month-old mice triggered YFP expression restricted to the luminal epithelial lineage^34^ with low efficiency in virgin mice (Supplementary Fig. 2b–e). Consistent with our results in *K14-Cre*mice (Fig. 2f, g), we found negligible labeling (0.3%) of perilipin+ adipocytes at week 4 of involution (Fig. 2g, Supplementary Fig. 2f). Collectively these data demonstrate that, in contrast to the findings of previous studies^32,33^, transdifferentiation between epithelial and adipocyte lineages is not a significant mechanism of either epithelial production during pregnancy or the re-establishment of mgWAT during involution.

### Identification of APs in murine and human MGs

**Fig. 3:**
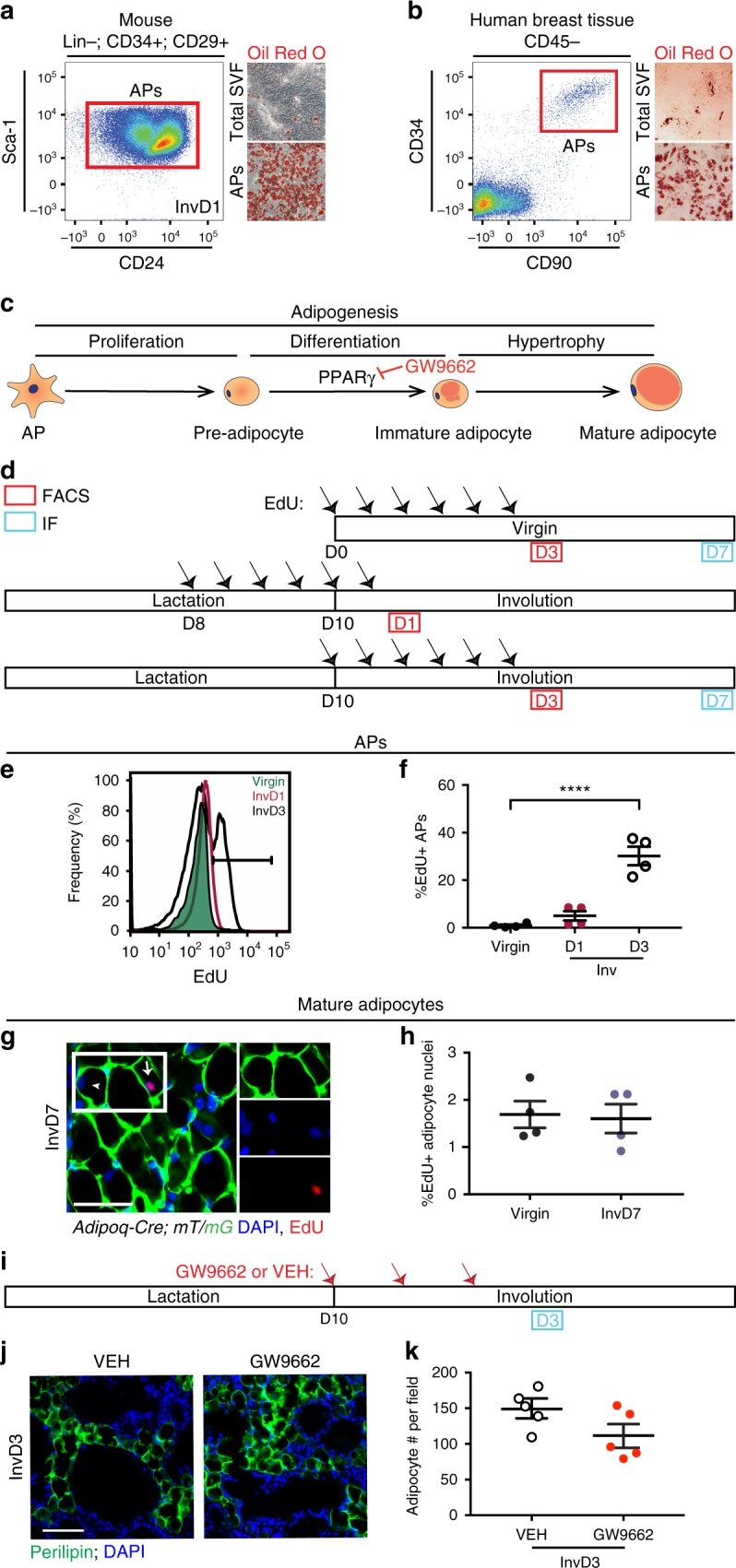
Evaluation of AP contribution to adipogenesis in mgWAT. **a**, **b** Representative flow cytometry plots of CD31−/CD45− (Lin−), CD34+, CD29+, Sca-1+, CD24± populations from the stromal vascular fraction (SVF) of fixed murine inguinal/mgWAT at involution day 1 (**a**, left panel) or CD45−, CD34+, CD90+ cells within the SVF of human breast tissue (**b**, left panel). Representative images of unpurified cells in total SVF or FACS purified adipocyte precursors (APs) from mice (**a**, right panel) or humans (**b**, right panel) after culturing in adipogenic media for 8 days (**a**) or 13 days (**b**) and staining with Oil Red O. *n* = 3 biological repeats. **c** Schematic summarizing stages of adipogenesis. **d** Experimental strategy to measure EdU incorporation in adipocyte precursors by FACS (as in **e** and **f**), and in mature adipocytes (as in **g**, **h**). **e** Representative flow cytometry plots of EdU signal in APs (as defined in **a**) in virgin mice and at involution days 1 and 3. **f** The average percentage of EdU+ APs identified with flow cytometry as in **e**. *n* = 4 mice per time point, *P* = 0.51 for virgin vs. involution day 1 data. **g, h** Representative image (**g**) and quantification (**h**) of EdU immunofluorescence in *Adipoq-Cre; mT/mG* mouse injected with EdU according to the timeline in **d**. Note EdU+ (arrow) and EdU− nuclei (arrowhead) in mGFP+ adipocytes. White box indicates insets. *n* = 200–800 nuclei in each of four mice per group, *P* = 0.84. **i** Experimental strategy to inhibit adipogenesis during involution using GW9662, a pharmacological antagonist of PPARγ. **j**, **k** Representative image (**j**) and quantification (**k**) of MGs from GW9662- and vehicle-treated mice, stained with a perilipin antibody and DAPI. *N* = 8–17 fields in each of five mice per treatment group, *P* = 0.09. Scale bar is 50 μm in **g** and **j**. Data are mean ± SEM. Statistics were performed using a one-way ANOVA with Tukey’s multiple comparisons test (**f**) and a two-tailed unpaired *t*-test (**h**, **k**). ****P < 0.0001. Lin lineage, PPARγ peroxisome proliferator-activated receptor gamma, Inv involution, D day, DAPI 4′,6-diamidino-2-phenylindole, EdU 5-ethynyl-2′-deoxyuridine, IF immunofluorescence, FACS fluorescence-activated cell sorting, SVF stromal vascular fraction

Another possible source of adipocytes are APs, which we and others have shown contribute to expansion of subcutaneous, visceral, and dermal WAT (sWAT, vWAT, and dWAT, respectively) by producing new mature adipocytes via adipogenesis^14–16,38^. To determine whether APs exist in MGs during involution, we enzymatically digested isolated MG tissue to generate a single cell suspension enriched for immune, endothelial, and mesenchymal cells called the stromal vascular fraction (SVF)^39^. We then analyzed the cells by flow cytometry for the AP marker profile^19,29,35,40^: Lineage− (Lin−, defined as CD45– and CD31−), CD34+, CD29+, Sca-1+, CD24±. A non-epithelial (Epcam−) cell population within the MG SVF expressed the markers of APs at involution day 1 (Fig. 3a and Supplementary Fig. 3a, b). When plated in culture and stimulated with adipogenic culture conditions, MG AP cells were able to differentiate into lipid-filled (Oil Red O+) adipocytes (Fig. 3a).

Next, we analyzed whether human MGs contain resident APs, which are CD45− and express CD34 and CD90 in human omental sWAT depots^41^. Flow cytometry analysis of SVF from reduction mammoplasty tissue revealed resident live, non-hematopoietic cells that express CD34 and CD90 (Fig. 3b and Supplementary Fig. 3c). FACS purification and culture of the CD45−, CD34+, CD90+ cells in adipogenic media displayed a ~3-fold enhancement of differentiation into lipid-filled mature adipocytes compared to total SVF or non-hematopoietic CD34−, CD90− stromal cells, as determined by Oil Red O lipid staining (Fig. 3b and Supplementary Fig. 3d).

Since several WAT depots have been shown to expand by adipogenesis in certain physiological scenarios^14–16,18,24^, we hypothesized that APs may also contribute to mgWAT expansion during involution (Fig. 1e). Complete adipogenesis—or the generation of new mature adipocytes—occurs in multiple steps: APs proliferate to generate pre-adipocyte progenitor cells, exit the cell cycle, terminally differentiate into mature adipocytes, and undergo hypertrophy as they fill with lipid (either by uptake or de novo synthesis)^14–16,19,27,28^ (Fig. 3c). To examine whether AP proliferation, the first step of adipogenesis, occurs during involution, we pulsed wild-type (WT) mice with EdU every 12 h for 3 days prior to analysis in virgin mice, or at involution days 1 or 3 (Fig. 3d). Few EdU+ APs were present in virgin or involution day 1 mice (0.9% and 5% of APs, respectively) (Fig. 3e, f). However, 30.2% of APs at involution day 3 were EdU+ (Fig. 3e, f), demonstrating that AP proliferation occurs in the early (reversible) phase of involution (Fig. 3f;  P≤ 0.0001 in comparison to virgin mice). This proliferative burst could reflect temporary or permanent expansion of the AP pool, or it could reflect the first stage of adipogenesis (AP proliferation, Fig. 3c) occurring in early involution.

### Long-lived mature adipocytes expand mgWAT during involution

Since APs proliferate during the first 3 days of involution, we reasoned that the majority of post-lactation expansion of mgWAT during the first 7 days of involution may occur via adipogenesis, in which case the differentiation and hypertrophy stages of adipogenesis would result in the formation of new mature cells (Fig. 3c). To determine whether proliferative APs produce new mature adipocytes during the first 7 days of involution, we pulsed virgin mice, or lactating mice upon initiation of involution, with EdU every 12 h for 3 days and chased for 4 days (Fig. 3d). We then analyzed whether mature, lipid-filled adipocytes contain EdU+ nuclei by immunofluorescence at day 7 of involution (Fig. 3g). Surprisingly, only 1.60% of visible nuclei within mature, lipid-filled adipocytes were EdU+ at involution day 7 (Fig. 3h). This low rate of EdU incorporation in adipocytes during involution is similar to the baseline level of incorporation observed in virgin mice (1.69%) (Fig. 3h; *P* = 0.84), and contrasts with the ~7% of nuclei that are labelled with BrdU in dWAT during the adipogenic response in which proliferative precursors form new mature adipocytes^16^.

As an additional approach to evaluate whether adipogenesis contributes to mgWAT expansion during early involution, we treated mice with GW9662, a PPARγ antagonist^42,43^ that inhibits adipogenesis in vivo^16,38,40^ (Fig. 3c). We administered GW9662 daily for the first 3 days of involution when adipocytes significantly repopulate the MG and APs proliferate (Fig. 3i). We examined mRNA levels in frozen mammary tissue, as well as sections immunostained with an antibody against perilipin (Fig. 3j). Consistent with inhibition of PPARγ, MGs from GW9662-treated mice demonstrated reduced expression of several late adipogenic and mature adipocyte-associated target genes of PPARγ^30,44,45^ (Supplementary Fig. 3e). However, in contrast to the abrogated adipogenesis observed in dWAT and bone marrow adipose depots following treatment with a PPARγ inhibitor^16,38,40,46^, an average of 112 adipocytes per frame were detected in mice treated with GW9662, which is similar to the average adipocyte number per frame (149) in vehicle-treated mice (Fig. 3k; *P* = 0.09). Thus, mgWAT expansion can occur in a largely PPARγ-independent manner.

**Fig. 4:**
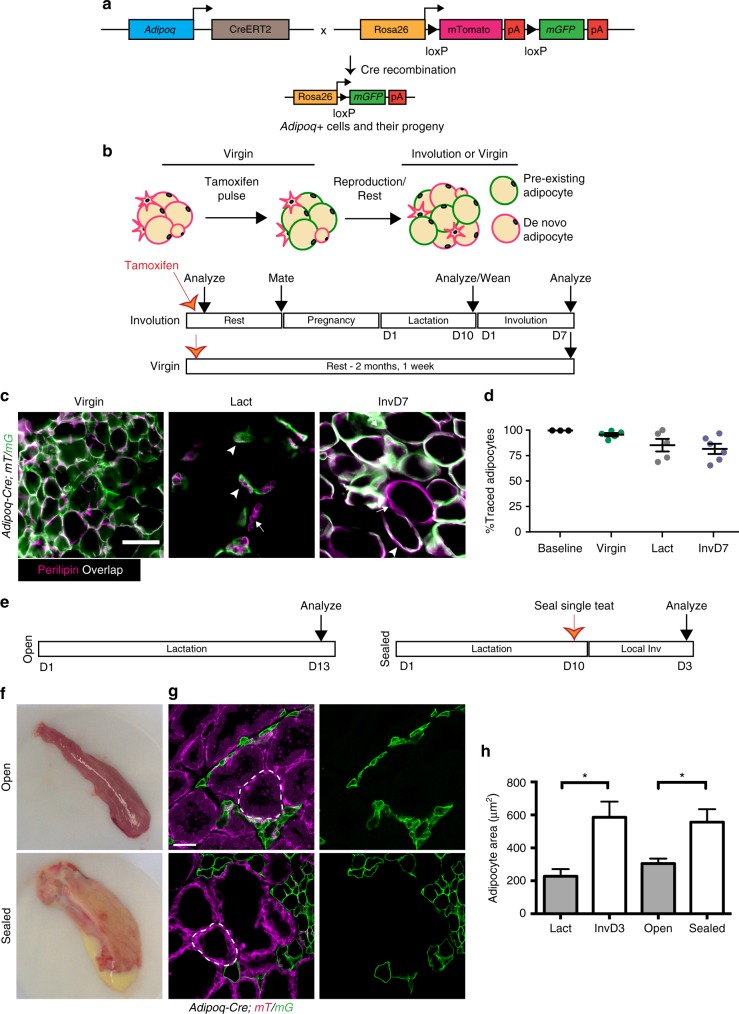
Hypertrophy is the major mechanism of mgWAT expansion during involution. **a** Schematic summarizing genetic strategy to induce mGFP expression in *Adipoq*-expressing cells and their progeny upon tamoxifen application. mTomato is expressed in all other cells. **b** Experimental scheme for in vivo analysis of adipogenesis during involution using *Adipoq-CreER; mT/mG* mice. Mice were treated with tamoxifen and analyzed at indicated time points. **c**, **d** Representative images (**c**) and quantification (**d**) of adipocyte lineage tracing at baseline (2 days post-tamoxifen pulse, image in Supplementary Fig. 4c), lactation day 10, involution day 7, or in virgin mice after 2 months in *Adipoq-CreER; mT/mG* mice following tamoxifen application. Arrowheads indicate traced (mGFP+) adipocytes, while arrows indicate untraced (mGFP−) adipocytes. *n* = 3171–20,020 adipocytes analyzed in 3, 5, 5, and 6 mice for baseline, virgin, lactation, and involution day 7 groups, respectively. **e** Experimental scheme for teat sealing assay, in which a single teat was sealed at day 10 of lactation to initiate a localized involution, while remaining teats were left open to continue unperturbed lactation. MGs were analyzed 3 days later. **f** Images of MGs with open teats or sealed teats. **g**, **h** Images of sections (**g**) and quantification of mGFP+ adipocyte cross-sectional area (**h**) in MGs from *Adipoq-Cre; mT/mG* mice with sealed or open teats. Dotted lines indicate examples of alveoli. Scale bars are 50 μm. Data are mean ± SEM. *n* = ~9000 adipocytes analyzed in six mice each for sealed and unsealed groups. Normal lactation and involution day 3 adipocyte area data are from Fig. 1h. Significance in **d** and **h** was calculated with a one-way ANOVA with Tukey’s multiple comparison test. *P < 0.05. D day, Inv involution, Lact lactation, *mT/mG* membrane tomato/membrane GFP

To further quantify the extent of adipogenesis during the lactation cycle, we examined the turnover of mature adipocytes using genetic lineage tracing with the adipocyte-specific, tamoxifen-inducible *Adipoq-Cre Estrogen Receptor* (*Adipoq-CreER*) mouse model crossed to the mT/mG reporter^15^ (Fig. 4a). While mGFP expression was minimal in adipocytes of untreated *Adipoq-CreER; mT/mG* mice (0.17% of perilipin+ cells expressed mGFP) (Supplementary Fig. 4a) or in APs from *Adipoq-Cre*; *mT/mG* mice (Supplementary Fig. 4b), application of a low dose of tamoxifen^35^ to *Adipoq-CreER; mT/mG* mice triggered a highly efficient (99.6%) switch from mTomato to mGFP in perilipin+, mature adipocytes within 48 h (Fig. 4b, d, Supplementary Fig. 4c).

To determine whether adipogenesis occurs in mgWAT during reproduction, we pulsed *Adipoq-CreER; mT/mG* mice with tamoxifen, mated the mice (Fig. 4b), and then quantified the percentage of mature adipocytes that formed de novo from mGFP-negative precursors after the tamoxifen pulse^15^ (Fig. 4c, mGFP− perilipin+ cells). Virgin mice were pulsed prior to at least 3 weeks of ‘rest’ to avoid known effects of tamoxifen on pregnancy and lactation^47,48^ (Fig. 4b). To ensure our detection of untraced (newly formed) mature adipocytes, we immunostained sections of MGs isolated from virgin, lactating, and involuting mice with antibodies against perilipin in combination with caveolin, which is expressed on the plasma membrane of mature adipocytes^15,16^ (Supplementary Fig. 4d). In virgin female mice, 95.5% of mature adipocytes were mGFP+ (Fig. 4c, d). During lactation and involution, over 80% of mature adipocytes maintained mGFP expression (Fig. 4c, d). The average percentage of adipocytes that were newly generated between lactation (14.8%) and involution day 7 (18.4%) were similar (Fig. 4d; *P* = 0.94).

Collectively, these experiments establish that a burst of AP proliferation occurs in early involution (Fig. 3e, f). Our data do not rule out the possibility that proliferative APs could complete the subsequent stages of adipogenesis (Fig. 3c) during involution. However, our experiments did not identify specific evidence pointing to the generation of a large number of newly formed adipocytes in the first 7 days post-weaning that might account for the radical growth of mgWAT during this time frame. Therefore, we focused our next experiments on evaluating how hypertrophy of mature adipocytes occurs.

### Adipocyte hypertrophy is not abrogated by lactation hormones

To investigate whether adipocyte hypertrophy can occur in the presence of lactation hormones, we performed teat sealing assays in *Adipoq-Cre; mT/mG* mice. Sealing single lactating MG teats results in milk stasis that induces a localized involution in the sealed MG, leaving the remaining unsealed (‘open’) teats functional as in a lactating MG, and preserving systemic lactation hormones^11^ (Fig. 4e). Although prolonged presence of lactation hormones prevents the collapse of alveolar ducts, central elements of the signaling that normally occur in early involution is mimicked in sealed teats: Stat5 activity is lost, while Stat3 phosphorylation, as well as transcription and translation of the death inducer *Bax* occurs, leading to normal initiation of programmed cell death^9,11^.

We sealed a single teat during lactation to stimulate localized involution, and after 3 days, analyzed both the MG associated with the sealed teat, and the MG associated with the contralateral teat that remained open for continued lactation (Fig. 4e). MGs associated with open teats were depleted of milk, while MGs associated with sealed teats displayed milk accumulation macroscopically (Fig. 4f) and epithelial lumens appeared distended in tissue sections, reflecting milk accumulation in alveoli prior to the collapse of these structures^11,49^ (Fig. 4g). MGs with an open teat contained small mGFP+ mature adipocytes (Fig. 4g) that were similar in size to mature adipocytes in lactating MGs (Fig. 4h). In contrast, mGFP+ adipocytes within the MGs attached to sealed teats were significantly larger than the mGFP+ adipocytes in MGs associated with open teats (on average 556 vs. 305 µm^2^, respectively), and were equivalent in size to adipocytes at involution day 3 (Fig. 4g, h), indicating that adipocytes undergo hypertrophy in response to involution-like changes, even in the presence of lactation-associated hormones.

### Epithelial-derived FAs traffic to mgWAT during involution

**Fig. 5:**
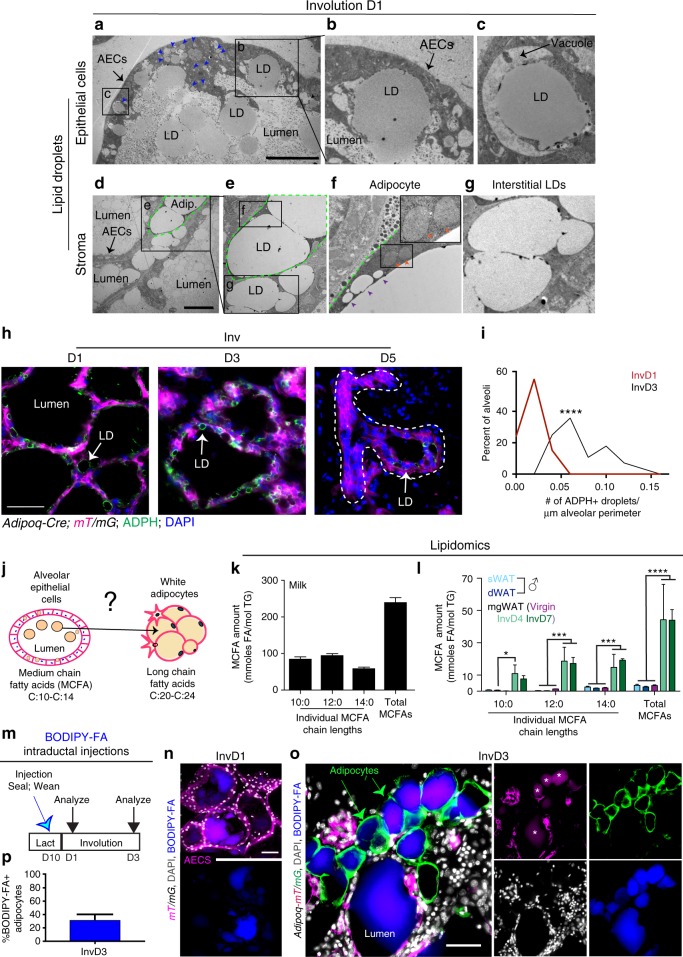
Epithelial-derived milk lipid is transferred to adipocytes during involution. **a**–**g** MG ultrastructure at involution day 1 examined by TEM. **a** LDs in alveolar lumen and within AECs (blue arrowheads). Enlarged micrographs of boxed regions in **a** containing LD **b** localized in apical AEC membrane and **c** associated with vacuoles. **d** Lipid outside of three alveoli, in interstitial space, and within adipocyte (dotted green outline). Boxed region of **d** enlarged in **e**. Boxed regions in **e** further enlarged to show **f** adipocytes with visible mitochondria (inset, orange arrowheads), small LDs (purple arrowheads), and **g** extracellular LDs in interstitial space. TEM data are representative micrographs from two mice. **h** MG cross sections from *Adipoq-Cre; mT/mG* mice at involution days 1, 3, and 5, immunostained with ADPH antibodies. Arrows indicate examples of ADPH+ LDs, dotted line traces remodeling alveoli. **i** Distribution of ADPH+ droplet number per μm of alveolar duct perimeter at involution days 1 and 3. Difference between # of ADPH+ droplets in 28–33 total ducts in three mice per time point was calculated using a two-tailed unpaired *t*-test. **j** Schematic of lipidomics experiment to evaluate AEC-produced MCFAs and adipocyte-produced LCFAs in adipocytes. **k**, **l** Average quantity of specific and total MCFAs from milk (**k**) and each WAT depot indicated (**l**). Significance calculated using two-way ANOVA with Tukey’s multiple comparison test. *n* mice = 6 (sWAT), 5 (dWAT), 3 (milk), 4 (mgWAT at involution day 4), and 3 (mgWAT at other time points). **m** Timeline to track BODIPY-FA. **n–p** Representative images of BODIPY-FA localization in *Adipoq-Cre; mT/mG* mice at involution days 1 (**n**) and 3 (**o**), and quantification of the percentage of adipocytes containing BODIPY-FA at involution day 3 (**p**). Asterisks indicate background. *n* = 3 biological repeats per time point. Scale bars are 20 μm (**a**, **d**, **h**), 50 μm (**n**), and 100 μm (**o**). Data in **p** is mean ± SD. *P < 0.05, ***, *P* < 0.001, ****P < 0.0001. TEM transmission electron microscopy, AECs alveolar epithelial cells, LD lipid droplet. Adip. adipocyte, ADPH adipophilin, MCFAs medium chain fatty acids, LCFAs long chain fatty acids, s/d/mgWAT subcutaneous/dermal/mammary gland white adipose tissue, BODIPY-FA boron–dipyrromethene–fatty acid

Given the significant increase in adipocyte size that occurs during the first week of involution, we next explored how adipocyte hypertrophy is driven during involution. Specifically, as large amounts of surplus milk fat fill MG epithelial lumens during post-weaning milk stasis, we considered whether MG adipocytes absorb remaining milk fats. In early involution, secretory AECs take up milk lipids from the alveolar lumen which initiates epithelial cell death and alveolar regression^50^. Consistent with this process, ultrastructural analysis by transmission electron microscopy (TEM) of MGs on day 1 of involution confirmed that milk lipid droplets were in both AECs and alveolar lumens (Fig. 5a), often embedded in the AEC apical membrane (Fig. 5a, b) or adjacent to vacuoles within AECs (Fig. 5a, c).

At this stage of involution, lipid droplets were also detected within adipocytes, which could be distinguished from free floating lipid by the presence of membrane-bound organelles including mitochondria (Fig. 5d, f). In contrast to the large, unilocular lipids typically within white adipocytes at a steady state, we noted adipocytes that harbored small lipid droplets immediately adjacent to large lipid droplets (Fig. 5e, f). This multilocular droplet presentation is indicative of lipid flux—either the maturation of a unilocular lipid droplet or its breakdown^51,52^. TEM also revealed extracellular lipid droplets in the interstitial space between adipocytes and ducts (Fig. 5d, e, g), in close proximity to adipocytes. The extracellular localization of these droplets was confirmed by the lack of membrane and organelles surrounding them.

Since extensive adipocyte hypertrophy occurs between 24 and 72 h after weaning (Fig. 1e, h), we examined the timing of lipid trafficking in mammary AECs during this time frame by immunostaining sections of MGs from *Adipoq-Cre; mT/mG* mice with an antibody against adipophilin (ADPH), a milk lipid-associated protein^53^. At 24 h into involution, ADPH+ droplets were observed inside alveolar lumen, within mTomato+ AECs, and occasionally in the interstitial space between alveolar ducts (Fig. 5h). The number of ADPH+ droplets in AECs increased significantly between days 1 and 3 of involution (Fig. 5h, i). By involution day 5, only a small amount of ADPH signal could be detected in some epithelial ducts (Fig. 5h). Thus, lipid trafficking during involution is concomitant with the first phase of MG adipocyte hypertrophy (Fig. 1e, h).

To determine whether milk lipids are taken up by adipocytes during involution as their lipid content increases (Supplementary Fig. 1c,d), we examined adipocyte FA content using lipid mass spectrometry. During lactation, AECs exclusively synthesize, package, and release medium chain fatty acids (MCFAs) of 10, 12, and 14-carbon lengths into alveolar lumens due to epithelial-specific expression of thioesterase II (OLAH, Uniprot ID Q8R197)^5,54^. Thus, we sought to determine whether MCFAs were present in adipocytes during involution (Fig. 5j). Adipocytes were isolated from several depots that have not been exposed to milk fat in order to control for the production and/or storage of MCFAs by adipocytes generally. We compared the lipid profiles from these depots with those from adipocytes derived from mgWAT from virgin female mice; as well as from mgWAT after lactation (involution days 4 and 7). Given diminished ADPH levels in epithelial ducts by involution day 5 (Fig. 5h), we did not expect that epithelial ducts would contain a substantial amount of residual lipid by involution day 7. However, to validate the purity of our adipocyte isolation method^16,55^, we confirmed that adipocyte isolates from involution day 7 samples were not contaminated with mGFP+ epithelial cells from *K14-Cre; mT/mG* mice (Supplementary Fig. 5a), and that minimal expression of epithelial-associated genes *Ep-CAM*, *K14*, and *K8* (Supplementary Fig. 5b) could be detected. The FA composition of total lipids was quantified and expressed as mmoles FA per mole of triglyceride^56^.

As expected, milk lipid was highly enriched for MCFAs (Fig. 5k), whereas adipocytes from virgin mgWAT, and male dWAT and sWAT contained small amounts of MCFAs (blue and magenta bars, Fig. 5l). Importantly, the absolute amount of MCFA present in adipocytes derived from MG at involution days 4 and 7 was significantly more than in control adipocytes (green bars, Fig. 5l), reaching levels nearly 20% of pure milk lipid. This observation is consistent with mgWAT taking up and storing AEC-derived FA during involution.

To test whether FAs in the lumen of MG alveoli are trafficked into mgWAT, we performed a fluorescent lipid tracking assay by direct intraductal injection of BODIPY-tagged fatty acid (BODIPY-FA)^57,58^ into the epithelial lumen of lactating mice (Fig. 5m). This technique allows localized delivery of materials to the MG epithelium^59,60^, as is evident in MGs from virgin and lactating mice after injection with Evan’s Blue vital dye (Supplementary Fig. 5c). We sealed the injected teats immediately after injection (Fig. 5m) to ensure retention of BODIPY-FA, and induced involution by weaning. 1 day post injection, BODIPY-FA was visible within the lumen of mTomato+ alveolar ducts of *mT/mG* mice (Fig. 5n). After 3 days, labeled lipid was clearly visible in ~38% mGFP+ adipocytes in 3 out of 3 *Adipoq-Cre; mT/mG* mice analyzed (Fig. 5o, p), demonstrating that adipocytes can take up lipid derived from epithelial lumen. BODIPY-FA was not visible in the livers of involution day 3 mice (Supplementary Fig. 5d), suggesting that a significant quantity of the FA analog was not in circulation. Taken together, these data reveal that as adipocytes undergo hypertrophy during involution, lipid accumulation in MG adipocytes is, at least in part, derived from milk lipids.

### Adipocytes are required for proper epithelial remodeling

**Fig. 6:**
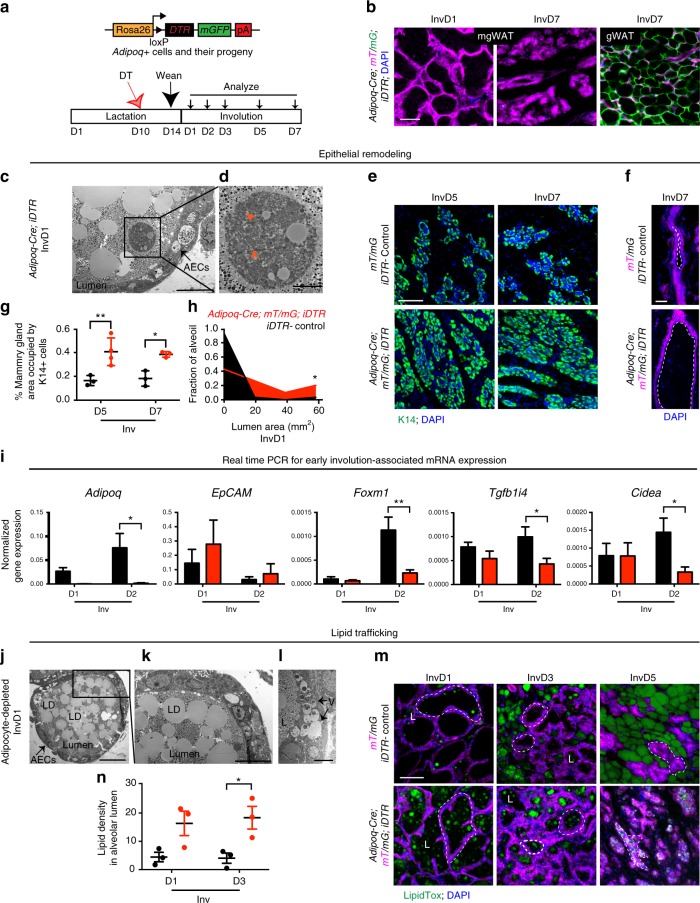
Adipocytes are required for proper lipid trafficking and epithelial remodeling. **a** Final genetic allele strategy (full strategy in Supplementary Fig. 6a) and timeline for mgWAT depletion. **b** mGFP+ adipocytes in mgWAT and gWAT at indicated time points post-mgWAT depletion, in DAPI-stained *Adipoq-Cre; mT/mG; iDTR* MGs. **c**, **d** Representative TEM images showing dead cell (boxed) in alveolar lumen in *mgWAT-depleted* mouse at involution day 1, enlarged in **d**. **e**, **f** Representative images of K14+ alveoli (**e**) and epithelial ducts (**f**) from *Adipoq-Cre; mT/mG; iDTR* and control mice. Dotted lines outline lumen. **g** MG area occupied by K14+ alveoli in *Adipoq-Cre; mT/mG; iDTR* and control mice from data in **e**. *n* = all visible alveoli in 5–10 frames, three mice per genotype. **h** Distribution of lumen area in epithelial ducts from *Adipoq-Cre; mT/mG; iDTR* and control mice from data in **f**. *n* = all visible epithelial ducts in entire 2D MG sections, three mice per genotype. **i** mRNA levels of early involution-associated genes in MGs from *Adipoq-Cre; mT/mG; iDTR* and control mice. Data are mean ± SEM (4–6 mice per genotype per time point). **j**–**l** Representative TEM images showing alveolar duct with dense LDs, visible in lumen (**j**), but not AECs (**k**), or adjacent to vacuoles (**l**). **m** Representative images of LipidTOX-stained MG sections from *Adipoq-Cre; mT/mG; iDTR* and control mice. Dotted lines outline examples of alveoli. **n** Average density of lipid in alveolar lumen at indicated time points, representing corrected total cell fluorescence of LipidTOX signal/luminal area. *n* = all alveoli in five 20× frames from three mice per genotype per time point. Significance was calculated using two-way ANOVA with Sidak’s multiple comparison test (**g**, **i**, **n**), or a two-tailed unpaired *t*-test comparing all ducts measured in depleted vs. control mice (**h**). *P < 0.05, **P < 0.005. Scale bars are 10 μm (**c, l**), 2 μm (**d**), 20 μm (**j**), 5 μm (**k**), and 100 μm (**b**, **e**, **f**, **m**). DT diphtheria toxin, H&E hematoxylin and eosin, TEM transmission electron microscopy, AECs alveolar epithelial cells, LDs lipid droplets, V vacuole, L lumen, K14 keratin 14, mgWAT and gWAT mammary gland and gonadal white adipose tissue

Based on our findings, we hypothesized that MG adipocytes may control lipid trafficking during involution. To test this hypothesis, we generated *Adipoq-Cre; mT/mG; inducible Diphtheria Toxin Receptor (iDTR)* mice, which allow temporal, site specific, and inducible deletion of mammary adipocytes via diphtheria toxin (DT) administration (Fig. 6a, Supplementary Fig. 6a). We injected DT directly into the 4th inguinal mgWAT depots of lactating mice prior to inducing involution (Fig. 6a), which induced specific near-total depletion of MG adipocytes prior to involution, as confirmed by the lack of mGFP+ adipocytes in the MG stroma in both early and late involution (Fig. 6b), and complete reduction of the perilipin signal (Supplementary Fig. 6b) that is normally present in the MG at this stage (Supplementary Fig. 1c, d). By contrast, adipocytes in gonadal WAT (gWAT), which is in close anatomical proximity to the treated inguinal mgWAT depot, and in dWAT, were still visible in mgWAT-depleted mice (Fig. 6b, Supplementary Fig. 6c). We also did not detect a difference between mgWAT-depleted and control mice in gWAT weight, or total mouse body weight (Supplementary Fig. 6d; *P* = 0.53–0.85 at each time point), suggesting that WAT depletion was most prominent in mgWAT. Tissue from DT-treated *Adipoq-Cre; mT/mG; iDTR* mgWAT-depleted mice, as well as control mice that were negative for either Cre or iDTR, was analyzed at involution days 1, 2, 3, 5, and 7 (Fig. 6a).

The lack of adipocytes in the MG stroma did not alter cell death events that normally occur during involution. Dead or dying cells in alveolar lumen were clearly visible in electron micrographs of *Adipoq-Cre; mT/mG; iDTR* mice, as could be distinguished by an abnormally dark cytoplasm (Fig. 6c) and evidence of organelle degradation including swollen mitochondria^61,62^ (Fig. 6d, orange arrowheads). Like control mice, MGs from *Adipoq-Cre; mT/mG; iDTR* mice also stained positively for indicators of involution-associated epithelial cell death at the expected time points, including phosphorylated Stat3, which regulates lysosomal-mediated cell death in epithelial cells during involution^63^, cleaved caspase 3, and TUNEL (Supplementary Fig. 6e). Despite these apparently normal aspects of the involution process, the remodeled alveoli in mice lacking MG adipocytes displayed altered organization. While the alveoli collapsed after involution day 3 in the absence of mgWAT (Supplementary Fig. 6f), a significant, ~2.5-fold increase in the percentage of the MG occupied by K14+ cells (Fig. 6e, g) was found in MGs lacking mgWAT by involution day 5, despite the similar 2D CSAs of MGs (<1% difference between means) at this stage (Supplementary Fig. 6g; *P* = 1.00). DT-treated *Adipoq-Cre; mT/mG; iDTR* mice also contained severely distended primary epithelial ducts at day 7 of involution in comparison to control mice (Fig. 6f, h), which was not observed in adipocyte-depleted virgin mice of the same genotype (Supplementary Fig. 6h). Furthermore, mRNA expression of genes that have been implicated in the regulation of epithelial programmed cell death in early involution^64^, and other mammary epithelial cell behaviors^65,66^ are significantly reduced in MGs of DT-treated *Adipoq-Cre; mT/mG; iDTR* mice compared to MGs of control mice (Fig. 6i). As expected, *Adipoq* mRNA levels were diminished in mgWAT-depleted glands, while the mRNA levels for the pan-epithelial gene *Ep-CAM* were similar between *Adipoq-Cre; mT/mG; iDTR* and control groups (Fig. 6i). Taken together, these data suggest that adipocytes also contribute to proper epithelial remodeling during early involution.

Given that adipocytes normally incorporate epithelial-derived lipid during involution (Fig. 5j–o), we wondered whether organizational defects in the remodeled epithelium (Fig. 6e–h) of adipocyte-depleted MGs might be associated with impaired lipid trafficking during involution. First, we examined lipid uptake by epithelial cells in adipocyte-depleted MGs by TEM as in mgWAT-normal mice in Fig. 5. We observed numerous alveolar ducts from *Adipoq-Cre; mT/mG; iDTR* mice that displayed an abnormally high density of lipid in the lumen (Fig. 6j) and unusual intracellular lipid localization in alveoli. Specifically, in the absence of mgWAT, we found that large lipid droplets were not bound to AEC apical membranes, fully encased by the cells (Fig. 6k), or connected to intracellular vacuoles within the cells (Fig. 6l), as was prevalent in MGs from unperturbed mice at this time point^50^ (Fig. 5a–c). *Adipoq-Cre; mT/mG; iDTR* MG tissue stained with H&E (Supplementary Fig. 6f) and the fluorescent neutral lipid stain LipidTOX confirmed that collapsed MG alveoli retained significantly elevated lipid concentration within their lumens throughout involution compared to control mice (Fig. 6m, n), pointing to a profound defect in lipid trafficking in the absence of stromal adipocytes. Collectively, these data demonstrate that adipocytes contribute to both epithelial remodeling and trafficking of lipid within alveoli during involution. Further studies are required to understand the precise relationship between lipid trafficking, epithelial organization, and mgWAT.

## Discussion

**Fig. 7:**
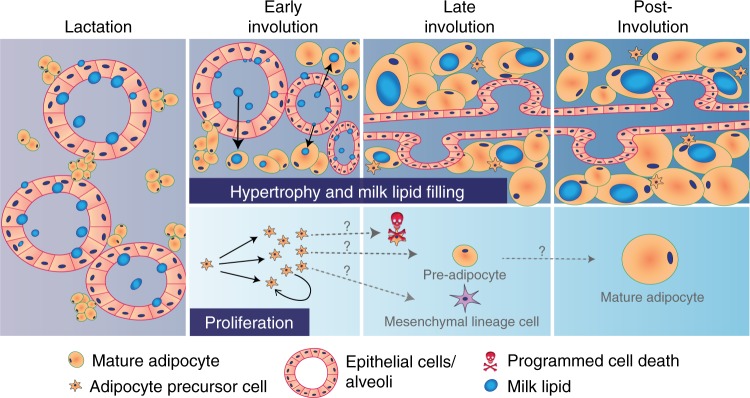
Model of adipocyte hypertrophy, lipid trafficking, and adipocyte precursor cell proliferation during lactation and involution. (Top) Mature adipocytes present in the mammary gland (MG) during lactation undergo hypertrophy and fill with milk lipids as epithelial alveoli regress during early involution. By late involution, a mammary fat pad consisting of long-lived adipocytes containing epithelial-derived lipid surrounds the remodeled epithelial ducts. (Bottom) Adipocyte precursors (APs) also proliferate during early involution. Several possible outcomes for newly formed cells during late involution include expansion of the AP pool in the MG, programmed cell death, or differentiation into other mesenchymal lineages. After involution, proliferative APs may commit to the adipocyte lineage and undergo adipogenesis to form new mature adipocytes

The cellular mechanisms underlying the adaptive responses of WAT to various physiological and pathological circumstances in support of tissue function is an area of increasing interest^24^. Here, we provide data demonstrating that while proliferative APs reside in the involution-stage MG, mgWAT growth during involution is largely due to hypertrophy of long-lived adipocytes coincident with the uptake of milk-derived lipid from epithelial lumens, as modeled in Fig. 7. A predominantly hypertrophy-driven mechanism of mgWAT enlargement during involution contrasts with mechanisms that expand adipose tissue via adipogenesis and hypertrophy in other adipose depots^14–16^. Instead, it more closely resembles the immediate refilling of pre-existing adipocytes in metabolic WAT depots that occurs in 1–4 days following starvation and refeeding^67^. Future studies examining other stromal cells or molecules that have been implicated in mgWAT repopulation, such as macrophages^68^ and ECM remodeling^69–71^, may reveal novel mechanisms that contribute to adipocyte hypertrophy or lipid trafficking during involution.

Several experiments presented here raise the intriguing possibility that adipogenesis may occur at stages of the lactation cycle before or after the first 7 days of involution. First, we show in genetic lineage tracing studies that in animals subjected to pregnancy, a small number of new adipocytes may be formed (~10% of mgWAT) before the 10th day of lactation (Fig. 4c, d). Second, as modeled in Fig. 7, we speculate that the AP proliferation observed at involution day 3 (Fig. 3f) may (1) contribute to other mesenchymal lineage cells such as fibroblasts during involution, which occurs in skeletal muscle^72^ or (2) produce new adipocytes after involution day 7 (Fig. 7). The latter possibility resonates with the timing of adipogenesis in response to high fat diet: AP proliferation is observed in the first week after high fat feeding, but newly formed adipocytes are observed 7 weeks following proliferation^15^.

Our data contrast with previous reports of interconversion between epithelial and adipocyte lineages during lactation cycles^32,33^ (Fig. 2f, g), which may reflect differences in lineage tracing models used. For example, the *aP2-Cre* mouse model used to trace mature adipocytes in the first report of reversible transdifferentiation^33^ was subsequently shown to label cells outside of the adipocyte lineage^36,37^, in contrast to the highly adipocyte- specific *Adipoq* promoter used in this study^30,36^. In addition, although cytoplasmic proteins, such as β-gal, were used as reporters to follow adipocytes in lineage tracing studies^33^, the localization of the reporter protein in the miniscule cytoplasm of mature adipocytes cannot be accurately interpreted. Increased precision of lineage relationships for adipocytes can be obtained with membrane-localized reporters such as the *mT/mG* protein used in our study^19,30,73,74^.

The adipocyte depletion experiments in this study extend prior work that establish lipid trafficking as a critical aspect of involution^50,85^. Stat3-controlled uptake of milk lipid from epithelial lumen was recently found to trigger leakage of lysosomes within epithelial cells, ultimately resulting in the initiation of epithelial PCD and alveolar collapse during involution^50^. We were surprised, therefore, to find evidence of epithelial PCD even in alveoli in which lipid uptake from the lumen appeared to be diminished. While this observation may reflect lipid uptake into AECs that is not completely abrogated in adipocyte-depleted mice, it may also suggest that Stat3 signaling, which is activated in adipocyte-depleted glands, can induce PCD, even when epithelial uptake of lipid is depressed. However, complete remodeling of epithelial cells and several genes associated with epithelial cell death are not fully induced in MGs lacking adipocytes (Fig. 6e–i), enforcing the idea that lipid uptake and/or the presence of mature adipocytes enable proper epithelial remodeling. The retention of lipid in the alveoli of adipocyte-depleted glands (Fig. 6m, n) may directly inhibit epithelial regression through unappreciated physical or molecular mechanisms. Adipocytes may also contribute to extracellular matrix proteins or inflammatory responses that are required for the re-organization of the mammary epithelial tree^66,70,76–78^. Further studies are needed to explore the mechanistic link between adipocyte behavior in the mammary stroma, lipid trafficking, and epithelial remodeling.

The role of lipid trafficking in tissue regulation is an emerging theme in epithelial biology. Our data reveal the previously unappreciated function of mature adipocytes in controlling lipid trafficking from the mammary epithelium. In addition to milk stasis and the stromal and immune factors discussed above, epithelial cells may reciprocally initiate adipocyte lipid dynamics since hair follicles can induce adipocyte lipid filling in the skin^17,25^ and mammary epithelial cells are known to stimulate lipolysis from adipocytes during lactation to help fuel the initial phase of milk production^1,5–7^. Lipid uptake by epithelial cells has been implicated also in melanoma, oral, and breast cancer metastasis^79^, and adipocyte-derived lipids promote ovarian cancer metastatic growth^80^. Given these connections between epithelial cells and adipocytes, and the fact that involution is inductive for pregnancy-associated breast cancer, our future studies will explore a role for lipid trafficking or other adipocyte-driven mechanisms in breast cancer progression. Since adipose tissue exists within several mammalian tissues^24,79^, the role of adipocytes in controlling lipid dynamics may have broad and profound implications for tissue homeostasis, regeneration, and disease.

## Methods

### Animals

All mouse studies were conducted according to the guidelines of Yale University’s Institutional Animal Care and Use Committee. The female mice were not randomized, and the investigators were not blinded to allocation during experiments and outcome assessment. For all studies of MG involution, offspring from the litter of first pregnancies were weaned between 9 and 11 days of lactation to induce involution. Involution day 1 represents 24 h following weaning. Litter sizes were normalized to 6–8 pups within each experiment by fostering and removing pups to/from the litter within a few days of birth.

Dual fluorescent *mT/mG* reporter mice in 129×1/SvJ or C57BL/6 backgrounds were purchased from Jackson Laboratories (stock nos. 007576 and 007676, respectively) and bred to the following strains: *Adipoq-Cre* in a C57BL/6 background (Jackson Labs stock no. 010803) for various studies to trace and deplete adipocytes and to perform fluorescent lipid tracking experiments; *Keratin 14-Cre in a CD1 background*, which were a generous gift from Elaine Fuchs (The Rockefeller University, New York, NY, USA) for transdifferentiation studies; *Adipoq-CreER* in a C57BL/6 background (Jackson Labs stock no. 024671) for lineage tracing of mature adipocytes; and *iDTR* in a C57BL/6 background (Jackson Labs stock no. 007900) for depletion of mature adipocytes. For lineage tracing of luminal epithelial cells, *K8-CreER* mice in a C57BL/6 background were generated by Cédric Blanpain’s Lab^34^ (Jackson Labs stock #017947), and crossed to the C57BL/6 *Rosa26-YFP* reporter mouse^82^ (Jackson Labs stock #006148). Adult CD1 wild-type mice (Charles River) were used to characterize development time points, evaluate APs with flow cytometry, and conduct GW9662 and teat sealing experiments.

For EdU (ThermoFisher Scientific, A10044) treatment, intraperitoneal injections (0.05 mg/g body weight) were administered twice daily; for GW9662 treatment, intraperitoneal injections (1 μg/g body weight) were administered daily for 3 days prior to experimental time points as previously described^40^; and for tracing with *K8-CreER; Rosa-YFP* mice, a single 1.0 mg peritoneal injection of tamoxifen diluted in sunflower oil was administered to 4-week-old female mice as was previously described^34^.

### Human samples

Human mammary stromal samples were either obtained at Duke University by Victoria Seewaldt under Duke Protocol Pro00011258 (P.I. Victoria Seewaldt) or under an exemption from the Yale University Human Investigations Committee to Matt S. Rodeheffer and Valerie Horsley. Both protocols underwent full review by either the Duke or Yale Institutional Review Board and informed consent was obtained from all subjects. For patients from Duke, subjects were recruited from the Duke Breast Clinics, and de-identified human adipose tissue obtained under Protocol Pro00011258 was provided by Victoria Seewaldt to Matt S. Rodeheffer and Valerie Horsley under an exemption from the Yale University Human Investigations Committee.

### Histology and immunostaining

Abdominal MGs (#4), located in the anterior sWAT depot, were dissected and fixed for 2 h in 3.2% paraformaldehyde (PFA) at room temperature, with the exception of MGs from *K8-CreER; Rosa-YFP* mice which were fixed in 4% PFA. MG tissue was then washed with PBS three times for 5 min each with shaking, and incubated overnight at 4 °C for 16 h in 30% sucrose diluted in PBS. MGs were then embedded in O.C.T. Compound (Tissue-Tek, 4583), frozen on dry ice, and stored at −80 °C. 14 μm thick cryosections were obtained using a Leica CM 3050S cryostat. Cryosections containing the region spanning the lymph node of the MG was used for all analyses.

For immunofluorescence (IF), sections were first incubated in blocking buffer (PBS containing 0.02% Gelatin, 0.25% Triton-X, 1% BSA, and normal donkey serum) for 1 h at room temperature. Primary antibodies diluted in blocking buffer were incubated overnight at 4 °C. The following primary antibodies were used: anti-GFP at 1:300 (chicken, Abcam, ab13970), anti-perilipin A at 1:1000 (goat, Abcam, ab61682), anti-cytokeratin 8 at 1:100 (rabbit, Abcam, ab53280 and chicken, Abcam, ab14053), anti-caveolin1 at 1:300 (rabbit, Cell Signaling, 3238), anti-adipophilin at 1:100 (guinea pig, Fitzgerald, 20R-AP002), anti-phospho-Stat3 at 1:100 (Tyr705, D3A7, rabbit, Cell Signaling, 9145), and anti-active caspase 3 at 1:100 (rabbit, R&D systems, AF835). Anti-cytokeratin 14 antibodies against the C-terminal sequence KVVSTHEQVLRTKN were generated by New England Peptide. A 10 min permeabilization with 100% methanol, followed by a 15 min PBS wash preceded incubation with anti-phospho-Stat3 antibodies. Following incubation with primary antibodies, slides were washed with PBS, and incubated with fluorescently tagged secondary antibodies (Jackson ImmunoReseach) against the appropriate species (diluted 1:250) for 1 h at room temperature. When indicated, slides were then washed with PBS for 15 min, and incubated for 20 min with TO-PRO 3 Iodide (642/661) (Invitrogen, T3605). Finally, slides were mounted with Prolong Gold, with or without DAPI (Invitrogen, P36935, P36934, respectively).

For histological analysis, tissue sections were stained with hematoxylin and eosin (H&E) according to standard protocols, and mounted with Fisher Chemical Permount Mounting Medium (ThermoFisher Scientific, SP15). For EdU imaging, slides were processed with the Click-iT EdU kit according to the manufacturer’s instructions (Invitrogen, C10337). For fluorescent lipid staining, slides were stained with LipidTOX Deep Red Neutral Lipid Stain (ThermoFisher Scientific, H34477) diluted 1:200 in PBS for 30 min, and then washed with PBS for 30 min. Throughout lipid staining procedure, slides were maintained in a horizontal position on ice. TUNEL staining was performed using Click-iT® Plus TUNEL Assay for In Situ Apoptosis Detection, Alexa Fluor® 647 dye (ThermoFisher Scientific, C10619) according to the manufacturer’s instructions. Slides stained for EdU, LipidTOX, and TUNEL were mounted with Prolong Gold with DAPI.

Whole-mount preparation for 3D imaging by confocal microscopy for transdifferentiation studies was conducted as described previously^30^. Briefly, dissected MGs were sliced into small pieces and squeezed between a coverslip and slide, and mounted in Aqua-Poly/Mount (Polysciences, Inc., 18606) for imaging tile scans and analysis.

### Image acquisition, processing, and analysis

IF microscopy was conducted on a Zeiss Axio Imager equipped with AxioVision Software. Image data quantified in Fig. 1i was acquired on a Hamamatsu camera with a smaller field size than Fig. 3j. Confocal imaging was conducted on a Zeiss LSM 510 confocal built using a Zeiss AXIO Observer Z1 inverted microscope, using ZEN software (Zeiss). For 3D imaging by confocal, 2–3 tile scans containing 30–40 *z*-stacks were acquired with a 20x objective and optical section resolution of 1024 × 1024, for each of three mice at the indicated time points. Maximum intensity projections of the resulting images were generated using ImageJ Software (NIH).

For morphometric analysis, the cross-sectional area of individual adipocytes, epithelial ducts, or MG cross sections was measured using ImageJ Software. Lineage tracing image data was quantified in ImageJ and Adobe Photoshop software. For analysis of adipocyte-depleted mice, lipid density in epithelial lumen is the corrected total cell fluorescence of luminal LipidTOX signal divided by the luminal area, both measured in ImageJ. Epithelial ducts spanning more than one microscope frame were stitched together using the Grid/Collection Stitching Plugin^83^ for ImageJ.

### Transmission electron microscopy (TEM)

TEM was performed in the Yale School of Medicine Center for Cellular and Molecular Imaging Electron Microscopy core facility. 2 *Adipoq-Cre; mT/mG; iDTR* and 2 *Cre-* or *iDTR-* mice were treated with DT as described in adipocyte depletion experiments below. One day after weaning, mice were perfused with 20–30 ml of 4% PFA at room temperature according to standard protocols^84^. MGs were extracted from the mouse and cut into small pieces, approximately 1 mm^3^ in volume. Tissue pieces were fixed in 2.5% glutaraldehyde/2% PFA in 0.1 M sodium cacodylate buffer, pH 7.4, for 30 min at RT and 1.5 h at 4 °C, and then rinsed in sodium cacodylate buffer three times. Samples were then postfixed in 1% osmium tetroxide for 1 h, rinsed and en bloc stained in aqueous 2% uranyl acetate for 1 h followed by rinsing, dehydrating in an ethanol series to 100%, rinsing in 100% propylene oxide, infiltrating with EMbed 812 (Electron Microscopy Sciences) resin, and baking overnight at 60 °C. Hardened blocks were cut using an ultramicrotome (UltraCut UC7; Leica). Ultrathin 60-nm sections were collected and stained using 2% uranyl acetate and lead citrate for transmission microscopy. Carbon-coated grids were viewed on a transmission electron microscope (Tecnai BioTWIN; FEI) at 80 kV. Images were taken using a CCD camera (Morada; Olympus) and iTEM (Olympus) software.

### Flow cytometry sorting and analysis

For flow cytometry of primary murine cells, MG or skin tissue was minced and digested in Hanks Balanced Salt Solution (HBSS) (Sigma, H8264) supplemented with 3% BSA, 0.8 mg/ml collagenase type 2 (Worthington Biochemical, LS004174), 0.8 mM ZnCl_2_, 1.0 mM MgCl_2_ and 1.2 mM CaCl_2_ for 75 min at 37 °C in a shaking water bath. Samples were shaken vigorously by hand for 1 min after 1 h of digestion. The resulting solution was then filtered through a 40 μm filter. For all studies, SVF is defined as the total non-floating fraction of the MG following centrifugation that is digestible by the collagen IV solution above. Cells from the SVF were pelleted at 300×*g*, washed with HBSS buffer containing 3% BSA, and stained with primary antibodies on ice for 30 min. For isolation of APs, the following antibodies were used: CD45 APC-eFluor 780 at 1:5000 (eBioscience; 47-0451-80), CD31 PE-Cy7 at 1:500 (eBioscience, 25-0311-82), CD29 Alexa Fluor 700 at 1:200 (BioLegend, 102218), CD34 Alexa Fluor 647 at 1:50 (BioLegend, 119314), Sca-1 Pacific Blue at 1:250 (BD Biosciences, 560653), and CD24 PerCP-Cy5.5 at 1:100 (eBioscience, 45-0242-82). In some experiments, anti-Ep-CAM (BD Pharmington #552370), which had been directly conjugated using the Alexa Fluor 647 Monoclonal Antibody Labeling Kit (Invitrogen #A20186) according to the manufacturer’s instructions, was also used for analysis at 1:100. For detection of EdU, the SVF was fixed and processed with the Click-iT EdU Alexa Fluor 647 Flow Cytometry Assay Kit (ThermoFisher Scientific, C10419) according to manufacturer’s instructions, and stained for the AP-associated antibodies listed above. For analysis of mammary epithelial cells, the MG was digested and processed for analysis as described in published methods^85^. For analysis of epithelial cells, cells were stained with the CD45, CD31, CD29, CD24, and Ep-CAM antibodies listed above.

For FACS analysis and isolation of human APs, reduction mammoplasty breast tissue was digested mechanically by mincing, and then chemically in modified KRP buffer (supplemented with 0.8 mM ZnCl_2,_ 1 mM MgCl_2_, 1.2 mM CaCl_2_) containing 3% fetal bovine serum (Atlanta Biologicals, S11150) and 1 mg/ml collagenase type 2 (Worthington Biochemical, LS004174) for 75 min at 37 °C with constant shaking. The digested suspension was filtered through a 250μm nylon filter and centrifuged at 300×*g* for 3 min, after which floating adipocytes and supernatant were removed. The SVF was washed with KRP supplemented with 3% FBS, filtered through a 70 μm filter, pelleted at 300×*g* for 3 min and washed with KRP, then filtered through a 40 μm filter, and pelleted at 300×*g* for 3 min. The resulting SVF pellet was then stained with the following antibodies on ice for 15–30 min: anti-human CD45 Pacific Blue at 1:400 (Biolegend, 304022), anti-human CD34 APC at 1:200 (eBioscience, 17-0349-42), and anti-human CD90 Pe-Cy7 at 1:2000 (BD Biosciences, 561668).

Following antibody incubation, mouse or human cells were washed with their respective buffers, unfixed cell preparations were treated with Sytox Orange (Invitrogen, 1:100,000) or propidium iodide (PI) (SigmaAldrich, P4864) at 0.5 g/ml in order to exclude dead cells, and cells were sorted or analyzed using a FACS Aria III equipped with FACS DiVA software (BD Biosciences). Single cell populations were selected based on forward scatter (FSC) and side scatter (SSC), and dead cells that had taken up Sytox Orange or PI were excluded. Single cells were isolated or analyzed based on cell surface markers. Data was analyzed using FlowJo version X.0.7 (FlowJo) or FACSDIVA (BD) software.

### RNA extraction and real-time PCR

GFP+ cells from lactating *Adipoq-Cre; mT/mG* mice were sorted directly into Trizol LS (Invitrogen, 10296-028); total MG tissue from GW9662-treated and *Adipoq-Cre; mT/mG; iDTR* (and associated control) mice was flash frozen and subsequently thawed and resuspended in Trizol (Sigma, T9424). RNA extraction and purification was performed using the RNeasy mini kit (QIAGEN 74104) following manufacturer instructions. cDNA was generated using equal amounts of total RNA with the Superscript III First-Strand Synthesis System (Invitrogen 18080051) using Oligo dT per the manufacturer’s instructions. Real-time PCR was performed as previously described^16,19^ using SYBR green I Master mix (Roche 04887352001) on a LightCycler 480 (Roche). Primers for specific genes are listed in the Supplementary information section (Supplementary Table 1). Results were normalized to β-actin expression as described previously^16,19^.

### Adipocyte precursor cell differentiation

In vitro analysis of the differentiation capacity of isolated APs, Oil red O (ORO) lipid staining, and ORO imaging was conducted as previously described^16^. For quantification of lipid accumulation, ORO was extracted by lysis by adding 100% isopropanol with 4% NP40 substitute, IGEPAL CA-630 (SigmaAldrich, #18896) (300 μl per well of a 24 well plate added), followed by gentle agitation for 10 min at room temperature. A 100 μl volume of this suspension was then transferred to a 96-well plate to measure absorbance at 490–520 nm using a plate reader. The ORO absorbance of six wells was averaged for each group (total SVF, non-hematopoietic stromal, and CD45−, CD90+, CD34+ cells).

### *Adipoq-CreER; mT/mG* lineage pulse chase

8-week-old female Adipoq-CreER; mT/mG mice were given a single 1.5 mg intraperitoneal injection of tamoxifen (SigmaAldrich, T5648) dissolved in sesame oil, followed by a 3 week recovery. Experimental mice were then subjected to mating, and mice were euthanized for analysis after 10 days of lactation, or after 10 days of lactation followed by 7 days of induced involution. Homeostatic control mice were left undisturbed for the duration of the experiment, and killed for analysis at the same time as the involution day 7 group. MG tissue was fixed and processed as described above for IF, and stained with GFP, perilipin and caveolin antibodies. Traced adipocytes (mGFP+ cells containing perilipin) and untraced/newly generated adipocytes (mGFP− Caveolin+ cells containing perilipin) were counted using ImageJ software.

### Teat sealing assay

The teat sealing assay was performed as described by Li et al.^11^ in *Adipoq-Cre; mT/mG* mice, followed by IF, image acquisition, and image data analysis using ImageJ software as described above.

### Quantitative lipid mass spectrometry

Adipocytes were isolated as described previously^16,55^. Briefly, the MG was digested with collagenase type 2 mixture as described above for analysis of primary murine APs with flow cytometry. Released SVF cells were centrifuged, and floating cells isolated and washed with HBSS with 3% BSA. The adipocyte isolate was then applied to glass slides for visualization (for analysis of purity in *K14-Cre; mT/mG* mice), frozen in TRIzol for subsequent RNA extraction and real-time PCR as described above, or frozen for mass spectrometry. Milk and adipose total lipids were extracted and FA profiles quantified by GC/MS as previously described^56,86^; data are expressed in millimoles of FA per mole of triglyceride in milk or adipose (mmoles FA/mol TG).

### Fluorescent lipid tracking assay

Pups were separated from lactating dams for 1 h, returned for 30 min, separated for 2 h, and returned for 15 min immediately prior to performing intraductal injection. 10 μl of BODIPY® FL C12 (4,4-difluoro-5,7-dimethyl-4-Bora-3a,4a-diaza-s-indacene-3-dodecanoic Acid) (ThermoFisher Scientific, D3822) diluted to 1.5 mg/ml with sesame oil, vehicle, or Evans Blue diluted in PBS was injected into teats of virgin or lactating mice. Intraductal injections have been described previously^59,60^; briefly, the tips of MG teats were clipped, and a 30 gauge beveled metal hub needle (Hamilton, 7748-16, 0.5 inch custom length needle) was inserted into the teat to inject needle content. Teats were sealed after injection, and pups were weaned to induce involution. MGs were extracted from mice at the time points indicated in Fig. 5m, fixed, frozen, cryosectioned, immunostained, and imaged as described above.

### Adipoq-Cre; mT/mG; iDTR adipocyte depletion

Female mice were subjected to mating and lactation. To deplete adipocytes, at lactation day 8–11, 400 ng of Diphtheria Toxin (SigmaAldrich D0564) was administered directly to the fat pads of abdominal MGs (#4) of *Adipoq-Cre; mT/mG; iDTR* mice or control mice (possessing either Cre− or iDTR− alleles). After a 4 day chase, pups were weaned to induce involution, and mgWAT, dWAT, and gWAT depots were harvested for cryopreservation and subsequent analysis at the time points indicated in Fig. 6a. Tissue preservation for electron microscopy or IF, image acquisition, and image data analysis using ImageJ software was performed as described above.

### Statistical analysis

No statistical method was used to predetermine sample size. Statistical tests used for each analysis and sample size for each group are indicated in figure legends. Statistical analysis was performed using GraphPad Prism 6. *P* < 0.05 was considered statistically significant.

## Electronic supplementary material

Supplementary Information

## Data Availability

All data generated or analysed during this study are included in this published article (and its supplementary information files).
