# Supplementary material for: Adipocyte hypertrophy and lipid dynamics underlie mammary gland remodeling after lactation
Source: Nat Commun. 2018 Sep 4;9:3592. doi: 10.1038/s41467-018-05911-0 (PMC6123393; doi:10.1038/s41467-018-05911-0)
Supplement: Supplementary file 1 — Supplementary Information [file 41467_2018_5911_MOESM1_ESM.pdf]

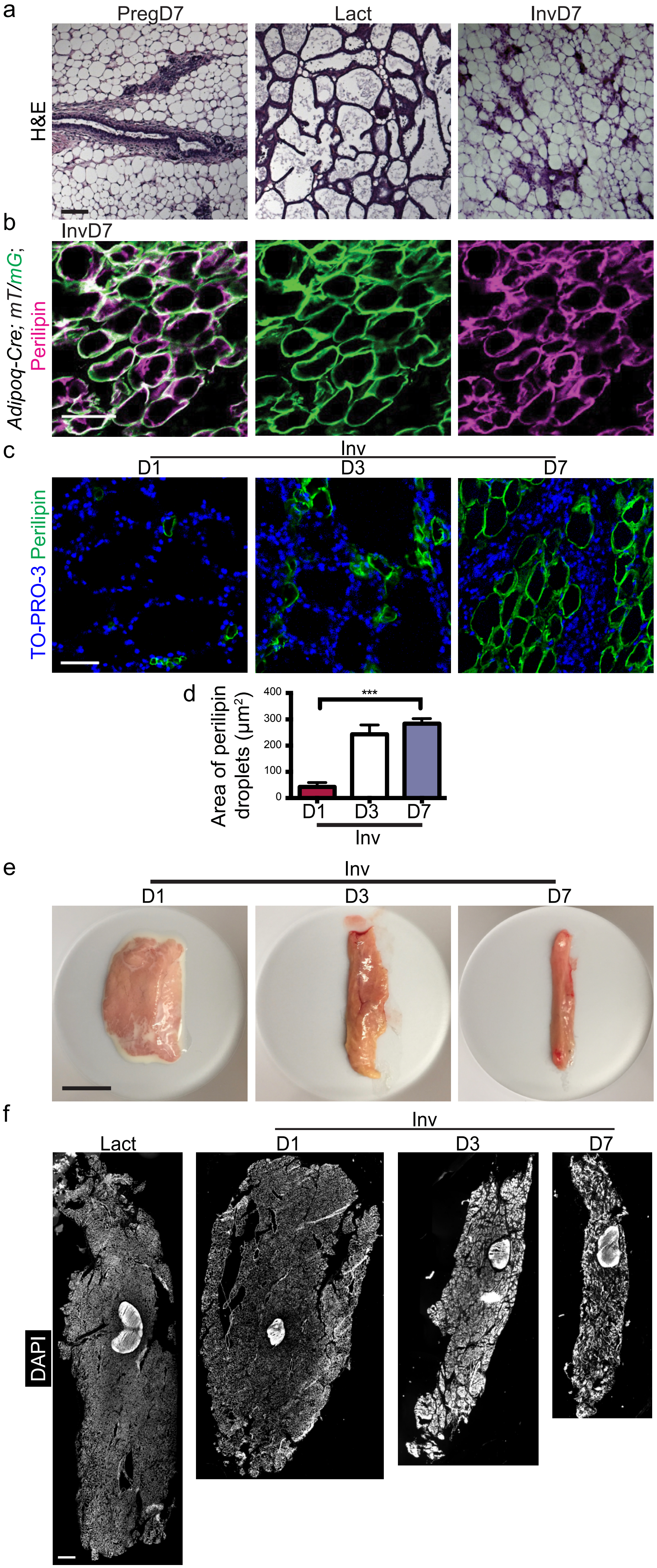

a
PregD7
Lact
InvD7
H&E
b
InvD7
Adipoq-Cre; mT/mG; Perilipin
c
Inv
D1
D3
D7
TO-PRO-3 Perilipin
d
Area of perilipin droplets (μm²)
400
300
200
100
0
***
D1
D3
D7
Inv
e
Inv
D1
D3
D7
f
Inv
Lact
D1
D3
D7
DAPI

Supplementary Figure 1. Analysis of adipocyte repopulation in MG stroma. (a) Representative images of MG sections stained with hematoxylin and eosin (H&E) at pregnancy day (D) 7, lactation (Lact) D10, and involution (Inv) D7. (b) Involution D7 MG from Adipoq-Cre; mT/mG mouse stained with perilipin antibodies. (c) Representative images of wild type mice stained for perilipin and nuclear stain TO-PRO-3 at involution days 1, 3, and 7. n=5 frames from each of 3 mice. (d) Average area of perilipin+ lipid droplets at involution days 1, 3, and 7. n=667-5,278 adipocytes quantified in 2-3 mice for each time point. Error bars represent mean ± SEM. Significance was calculated using one-way ANOVA with Tukey's multiple comparison test. ***(P<0.001). (e) Photographs of extracted MGs at involution days 1, 3, and 7. (f) Low magnification images of DAPI-stained mammary gland cross sections at lactation, and involution days 1, 3, and 7. Scale bars are 100 µm (a), 50 µm (b-c), 9 mm (e), or 1mm (f). DAPI: 4',6-diamidino-2-phenylindole.

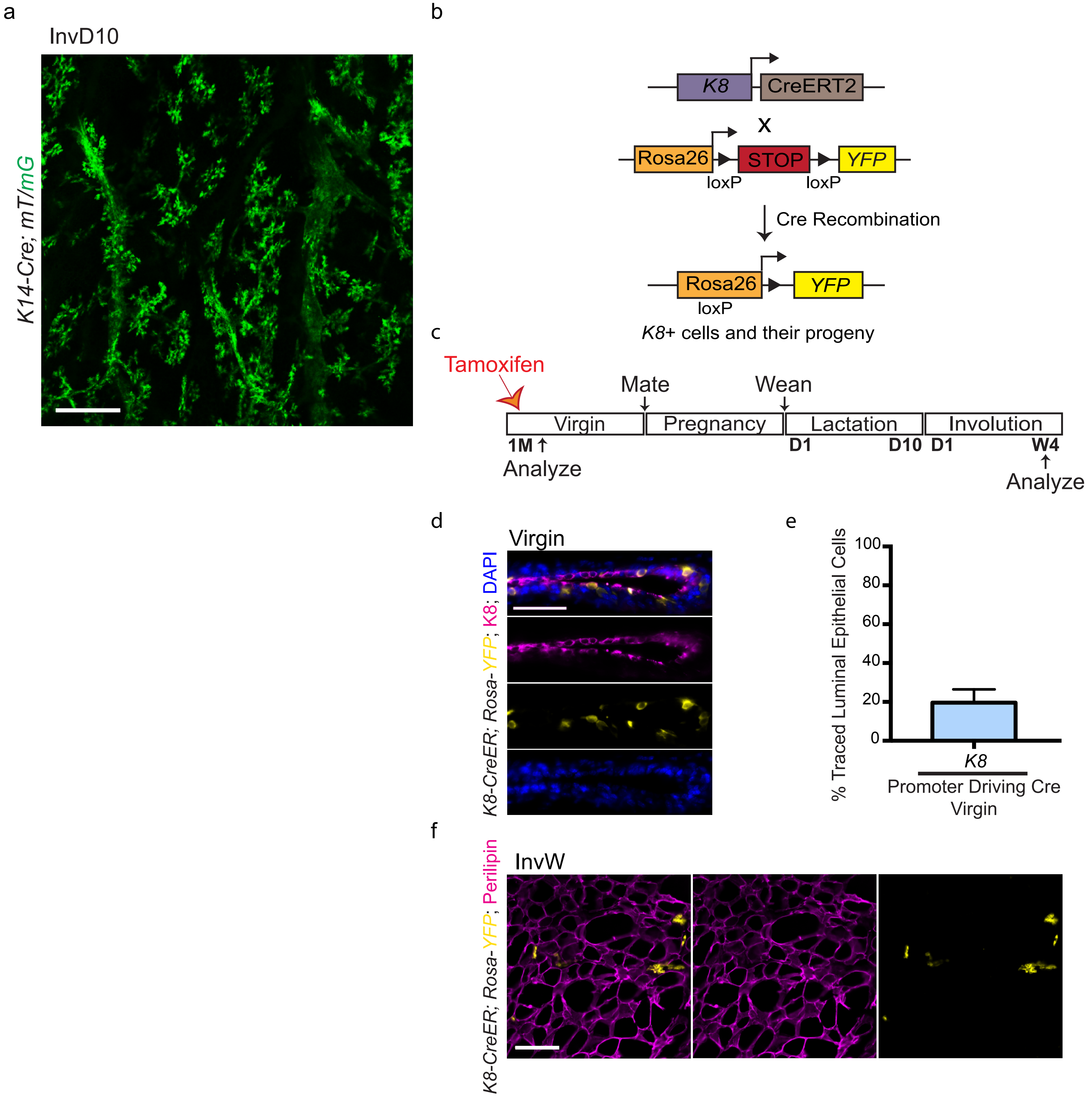

Supplementary Figure 2. Analysis of epithelial to adipocyte transdifferentiation during involution. (**a**) Whole mount MG tissue imaged at involution day 10 in *K14-Cre; mT/mG* mice. Three-dimensional projection of tile scan image representing 30-40 z-stacks. Scale bar is 500 µm. (**b-c**) Schematic summarizing genetic strategy (**b**) and experimental timeline (**c**) to drive YFP expression in K8-expressing cells and their progeny upon tamoxifen pulse. (**d**) MG cross sections from virgin *K8-CreER; Rosa-YFP* mice immunostained with antibodies against K8 and DAPI. (**e**) Quantification of the baseline percentage of YFP+, K8+ luminal epithelial cells in virgin *K8-CreER; Rosa-YFP* mice after tamoxifen pulse. n = 5 20x frames from 3 mice. Error bars represent mean ± SEM. (**f**) Representative image of MG from *K8-CreER; Rosa-YFP* mouse after involution (4 weeks after weaning), immunostained for perilipin. K: keratin, DAPI: 4',6-diamidino-2-phenylindole, Inv: involution, D: day, M: month, W: week.

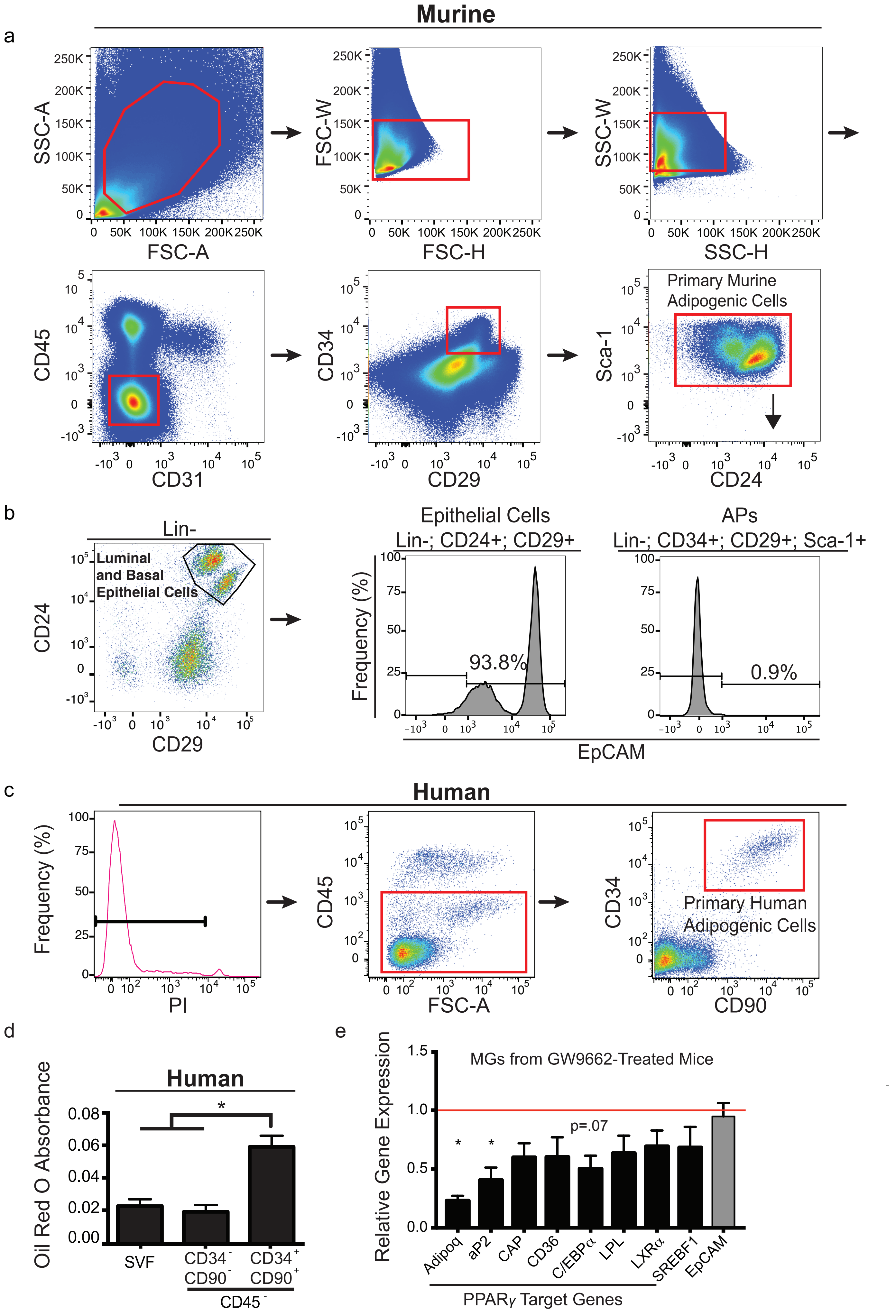
Murine
a
SSC-A
FSC-A
FSC-W
FSC-H
SSC-W
SSC-H
CD45
CD31
CD34
CD29
Sca-1
CD24
Primary Murine Adipogenic Cells
b
Lin-
Luminal and Basal Epithelial Cells
CD24
CD29
Epithelial Cells
Lin-; CD24+; CD29+
APs
Lin-; CD34+; CD29+; Sca-1+
Frequency (%)
93.8%
0.9%
EpCAM
c
Human
Frequency (%)
PI
CD45
FSC-A
CD34
CD90
Primary Human Adipogenic Cells
d
Human
Oil Red O Absorbance
*
SVF
CD34- CD90-
CD34+ CD90+
CD45-
e
MGs from GW9662-Treated Mice
Relative Gene Expression
p=.07
Adipoq
aP2
CAP
CD36
C/EBPα
LPL
LXRα
SREBF1
EpCAM
PPARγ Target Genes

Supplementary Figure 3. Analysis of APs in murine and human MGs. (a) Representative flow cytometry plots of sequential gating strategy for murine AP cells(CD45-, CD31-, CD34+, CD29+, Sca1 +, CD24+/-) in mgWAT at involution day 1. (b) Histogram of EpCAM expression in murine AP population defined in a. EpCAM expression in Lin- (CD45-, CD31-), CD24+ CD29+ luminal and basal epithelial cells serves as positive control. (c) Flow cytometry plots illustrating gating strategy and analysis of APs (CD45-, CD34+, CD90+) from human breast tissue (n=3 samples observed by flow cytometry). (d) Quantification of Oil Red O absorbance in total stromal vascular fraction (SVF), non-hematopoietic stromal cells (CD45-, CD34-, CD90-), and APs (CD45-, CD34+, CD90+), following culture in adipogenic media for 13 days and staining with Oil Red O as in Fig. 3b. n=6 wells for each group. (e) qPCR results showing average expression of adipogenic and mature adipocyte PPAR target genes, and an epithelial-associated gene (control), in MGs extracted from GW9662-treated mice, normalized to mRNA levels in MGs from vehicle-treated controls. n=5 mice per treatment group. *(P<0.05). FSC: forward scatter, SSC: side scatter: Lin: lineage, MG: mammary gland.

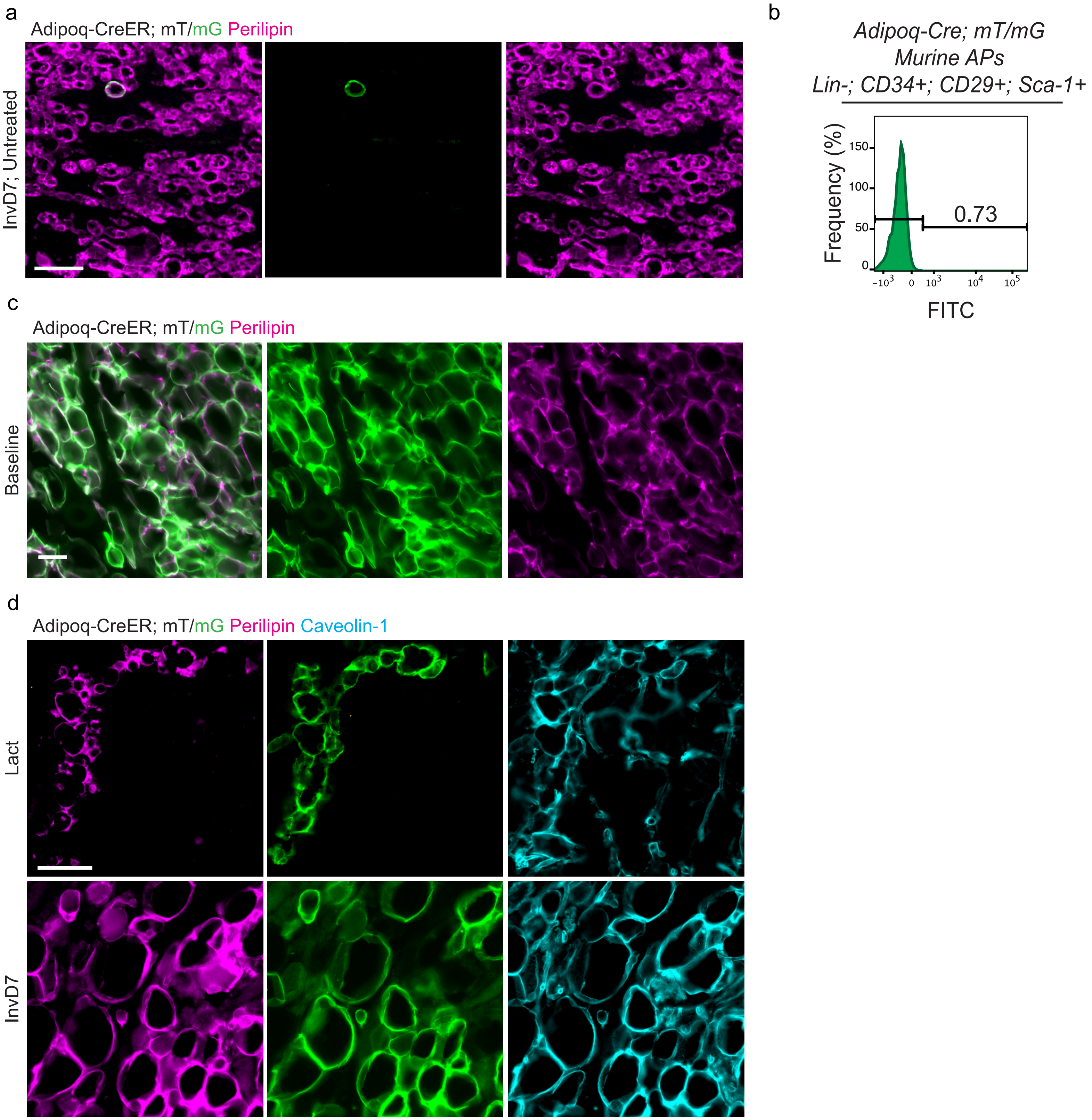

Supplementary Figure 4. ***Adipoq-Cre*** **activity in MG epithelium during involution.** (**a**) Representative images of *Adipoq-CreER; mT/mG* tissue in a mouse at involution day 7 that was not treated with tamoxifen. (**b**) Histogram of mGFP expression in murine APs as defined in Supplementary Fig. 3a, in *Adipoq-Cre; mT/mG* mice. n=3 mice. (**c**) MG tissue from *Adipoq-CreER; mT/mG* mice 2 days after tamoxifen application (baseline time point) stained with antibodies against perilipin. (**d**) Lactation (Lact)- and involution day 7 (InvD7)-stage MG tissue stained with antibodies against perilipin and caveolin to demonstrate strategy for visualizing mGFP+ and mGFP- mature adipocytes for quantification in pulse chase experiments in Fig. 4d. Scale bars are 50 μm in **a** and **c** and 100 μm in **d**.

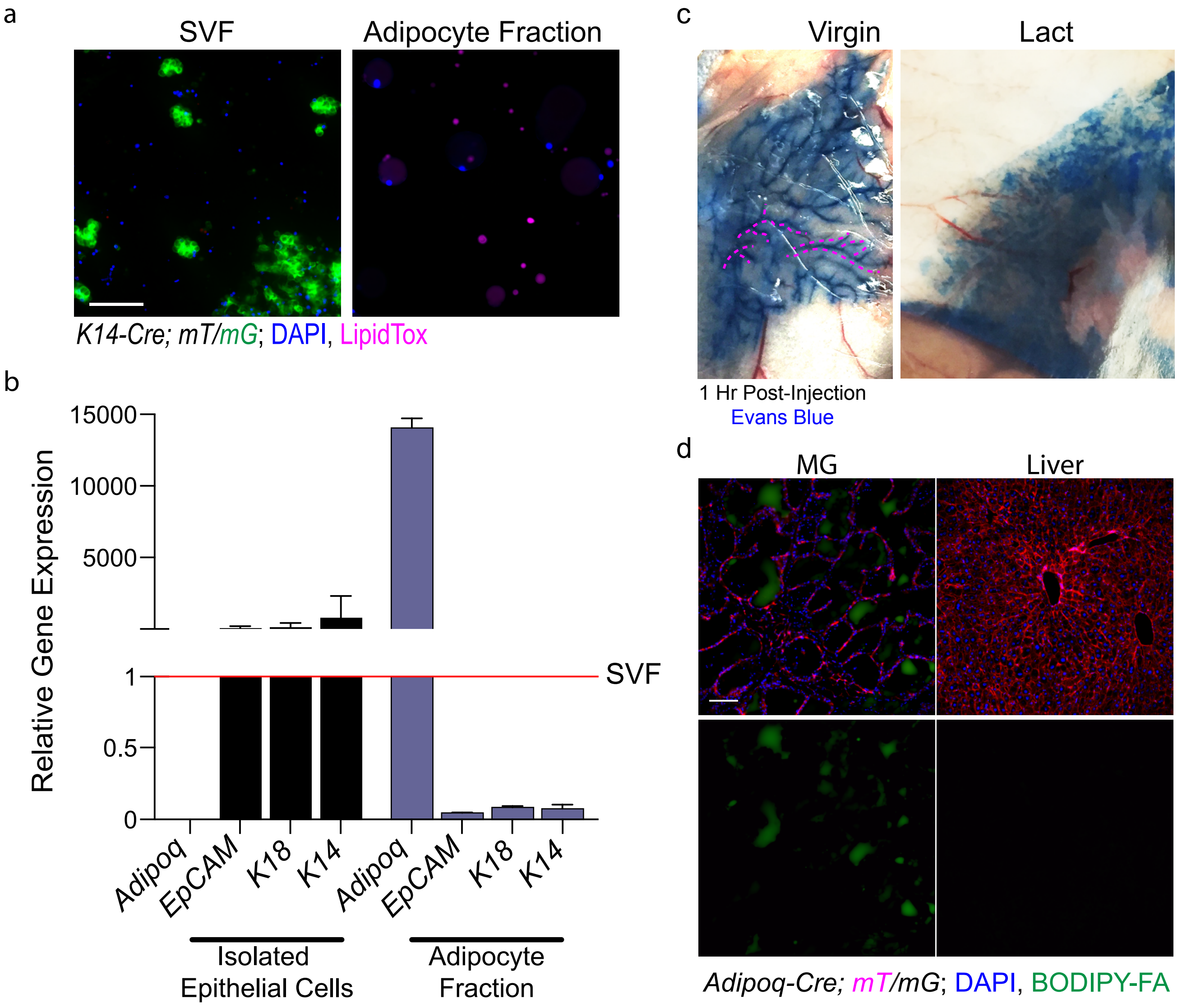

Supplementary Figure 5. Analysis of BODIPY-FA intraductal injections. (**a**) Representative images of cell suspensions from the stromal vascular fraction (SVF) and adipocyte fraction of *K14-Cre; mT/mG* mice stained with LipidTox and DAPI. Scale bar represents 100μm. (**b**) Representative qPCR results showing expression of mature adipocyte and epithelial-associated genes in isolated epithelial cells (positive control) and adipocyte fractions of the MG, normalized to total SVF (red line). n=3 biological repeats. (**c**) Intraductal injections of Evans Blue vital dye, performed in virgin and lactating wild type mice. Dotted magenta lines trace dye in one region of MG epithelium. (**d**) Representative images of BODIPY-FA signal in cross sections of MG and liver from an intraductally-injected *Adipoq-Cre; mT/mG* mouse stained with DAPI. SVF: stromal vascular fraction, Lact: lactation, K: keratin, DAPI: 4',6-diamidino-2-phenylindole, BODIPY-FA: boron-dipyrromethene-fatty acid.

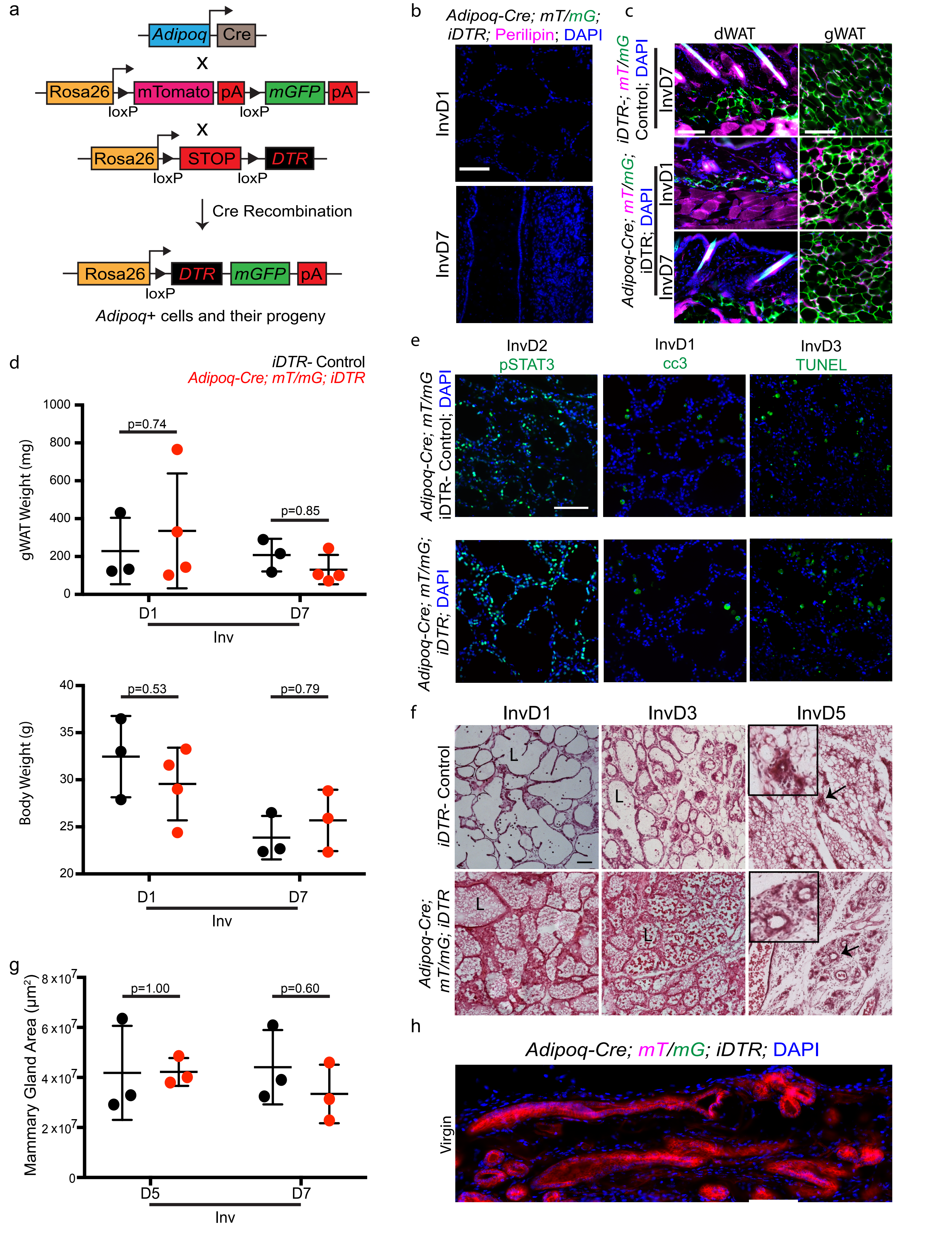

a
Adipoq
Cre
x
Rosa26
mTomato
pA
mGFP
pA
loxP
loxP
x
Rosa26
STOP
DTR
loxP
loxP
Cre Recombination
Rosa26
DTR
mGFP
pA
loxP
Adipoq+ cells and their progeny
b
Adipoq-Cre; mT/mG; iDTR; Perilipin; DAPI
InvD1
InvD7
c
dWAT
gWAT
iDTR-; mT/mG Control; DAPI
InvD7
Adipoq-Cre; mT/mG; iDTR; DAPI
InvD1
InvD7
d
iDTR- Control
Adipoq-Cre; mT/mG; iDTR
gWAT Weight (mg)
1000
800
600
400
200
0
p=0.74
p=0.85
D1
D7
Inv
Body Weight (g)
40
35
30
25
20
p=0.53
p=0.79
D1
D7
Inv
e
InvD2
pSTAT3
InvD1
cc3
InvD3
TUNEL
Adipoq-Cre; mT/mG iDTR- Control; DAPI
Adipoq-Cre; mT/mG; iDTR; DAPI
f
InvD1
InvD3
InvD5
iDTR- Control
Adipoq-Cre; mT/mG; iDTR
L
g
Mammary Gland Area (μm²)
8 x10^7
6 x10^7
4 x10^7
2 x10^7
0
p=1.00
p=0.60
D5
D7
Inv
h
Adipoq-Cre; mT/mG; iDTR; DAPI
Virgin

Supplementary Figure 6. Characterization of Adipoq-Cre; mT/mG; iDTR mice. (a) Schematic summarizing genetic strategy to drive mGFP and iDTR expression in Adipoq-expressing cells in order to ablate them upon local administration of diphtheria toxin. mTomato is expressed in all other cells. (b) Depletion of mGFP+ adipocytes at involution days 1 and 7 in Adipoq-Cre; mT/mG; iDTR mice, stained for DAPI and with antibodies against perilipin. (c) dWAT and gWAT from control and Adipoq-Cre; mT/mG; iDTR mice at indicated involution time points after DT injection and forced weaning. gWAT data from experimental mouse at involution day 7 is from Fig. 6b. (d) Quantification of gWAT (top) and total body (bottom) weights from Adipoq-Cre; mT/mG; iDTR and control mice at indicated involution time points after DT injection. n=3-4 mice per group per timepoint, p values indicated on graphs. (e) MG sections from Adipoq-Cre; mT/mG; iDTR and control mice from the indicated time points were stained as indicated for: phosphorylated Stat3, cleaved caspase 3, and TUNEL cell death staining. (f) Representative images of H&E-stained sections of MGs from Adipoq-Cre; mT/mG; iDTR mice and iDTR negative controls from the indicated time points. Black boxes indicate insets of regions of the tissue indicated by arrows. Alveolar lumen are labelled with ‘L’. (g) Average area of MG cross sections at the indicated time points. n=3 mice per group per timepoint, p values indicated on graph. (h) Representative image of MG tissue from DT-treated virgin Adipoq-Cre; mT/mG; iDTR mouse, stained with DAPI. Significance in d and g was evaluated by two-way ANOVA with Sidak’s multiple comparison test. DT: diphtheria toxin, dWAT and gWAT: dermal and gonadal white adipose tissue respectively, DAPI: 4’,6-diamidino-2-phenylindole, pStat3: phosphorylated Stat3, cc3: cleaved caspase 3.

Supplementary Table 1: Primer Sequence Information

Gene	Primer Sequence 5'-3'
Adipoq	**for** CACACCAGGCCGTGATGGCA<br>**rev** CAGTGACGCGGGTCTCCAGC
aP2	**for** GTCACCATCCGGTCAGAGAG<br>**rev** GCTCTTCACCTTCCTGTCGT
C/EBPα	**for** CCCAGAGGACCAATGAAATGAAG<br>**rev** TAGCCGGAGGAAGCTAAGAC
CAP	**for** TACATCGAAGGGGAGAAAGTGG<br>**rev** TCTTTATCATCGTGCCGTCTCC
CD36	**for** GGCCAAGCTATTGCGACATG<br>**rev** CCGAACACAGCGTAGATAGAC
Cidea	**for** ACAAGAGCCACCAACATCACCAAATCCT<br>**rev** CCGCGTCTCTTAAATCACTGCCCTATCA
EpCAM	**for** GCTGTCATTGTGGTGGTGTC<br>**rev** CACGGCTAGGATTAAGCTC
Foxm1	**for** CCCCGCCGGCCACTGATTCT<br>**rev** GGGCGTGGTGGGGTGGTTGATA
GFP	**for** TCTGCACCACCGGCAAGCTG<br>**rev** TGCGCTCCTGGACGTAGCCT
K14	**for** TCAGCATGAAAGCATCCCTGGAGAA<br>**rev** ATTTGGCGGCTGGAGGAGGTCA
K18	**for** TCCATCTGTGCCTTGTAT<br>**rev** GACGCTGAGACCACACT
Leptin	**for** GCTGGAGACCCCTGTGTCGGT<br>**rev** GCCAGTGACCCTCTGCTTGGC
LPL	**for** TGCGCCTCCTGCTCAAC<br>**rev** CTCGGAAGGCGGTCAAACT
LXRα	**for** GGCCGGCCGGGGAGGAGTTAGT<br>**rev** ACAGGGGCCAGCAGGGAGAAGCAGTA
Perilipin	**for** GACACCACCTGCATGGCT<br>**rev** TGAAGCAGGGCCACTCTC
PPARγ2	**for** TTCGCTGATGCACTGCCTATGAG<br>**rev** ACAGAGCTGATTCCGAAGTTGGTG
SREBF1	**for** GCCCCTGCCCACCTCAAACCTG<br>**rev** CACTGGCACGGGCATCCTTCCTCA
Tgfb1i4	**for** CCCCCGGTACCCAGCACAATG<br>**rev** GGAGCAGCGAAGGAGGAGGAAAATC
